# Active subnetwork recovery with a mechanism-dependent scoring function; with application to angiogenesis and organogenesis studies

**DOI:** 10.1186/1471-2105-14-59

**Published:** 2013-02-21

**Authors:** Ilana Lichtenstein, Michael A Charleston, Tiberio S Caetano, Jennifer R Gamble, Mathew A Vadas

**Affiliations:** 1 School of Information Technologies, University of Sydney, Sydney, NSW 2006, Australia; 2 Sydney Emerging Infections and Biosecurity Institute, University of Sydney, Sydney, NSW 2006, Australia; 3 Centre for Mathematical Biology, University of Sydney, Sydney, NSW 2006, Australia; 4 NICTA, Australian Technology Park, Eveleigh, NSW 2015, Australia; 5 Research School of Computer Science, Australian National University, Canberra, ACT, 0200, Australia; 6 Vascular Biology Laboratory, Centenary Institute, Camperdown, NSW 2050, Australia; 7 Sydney University Medical School, University of Sydney, NSW 2006, Australia

**Keywords:** Condition-specific networks, Active subnetwork, Markov random field, Edge label

## Abstract

**Background:**

The learning active subnetworks problem involves finding subnetworks of a bio-molecular network that are active in a particular condition. Many approaches integrate observation data (e.g., gene expression) with the network topology to find candidate subnetworks. Increasingly, pathway databases contain additional annotation information that can be mined to improve prediction accuracy, e.g., interaction mechanism (e.g., transcription, microRNA, cleavage) annotations. We introduce a mechanism-based approach to active subnetwork recovery which exploits such annotations. We suggest that neighboring interactions in a network tend to be co-activated in a way that depends on the “correlation” of their mechanism annotations. e.g., neighboring phosphorylation and de-phosphorylation interactions may be more likely to be co-activated than neighboring phosphorylation and covalent bonding interactions.

**Results:**

Our method iteratively learns the mechanism correlations and finds the most likely active subnetwork. We use a probabilistic graphical model with a Markov Random Field component which creates dependencies between the states (active or non-active) of neighboring interactions, that incorporates a mechanism-based component to the function. We apply a heuristic-based EM-based algorithm suitable for the problem. We validated our method’s performance using simulated data in networks downloaded from GeneGO against the same approach without the mechanism-based component, and two other existing methods. We validated our methods performance in correctly recovering (1) the true interaction states, and (2) global network properties of the original network against these other methods. We applied our method to networks generated from time-course gene expression studies in angiogenesis and lung organogenesis and validated the findings from a biological perspective against current literature.

**Conclusions:**

The advantage of our mechanism-based approach is best seen in networks composed of connected regions with a large number of interactions annotated with a subset of mechanisms, e.g., a regulatory region of transcription interactions, or a cleavage cascade region. When applied to real datasets, our method recovered novel and biologically meaningful putative interactions, e.g., interactions from an integrin signaling pathway using the angiogenesis dataset, and a group of regulatory microRNA interactions in an organogenesis network.

## Background

One aim of systems biology is to map high-throughput OMICs data or a list of genes to known pathways, as well as discover novel networks of interactions. Advanced integrated platforms such as Metacore from GeneGo Inc, Ingenuity Pathway Analysis (Ingenuity Systems, http://www.ingenuity.com), or GeneSpring Pathway Analyzer (Agilent Technologies) provide tools to allow such discoveries by mining large proprietary, often manually annotated, databases for networks of interactions enriched for genes/proteins in the user’s input gene list. The details relating to each interaction are supported by evidence in scientific literature. Such details allow the interactions to be categorized in different ways, such as effects (positive, negative, or “other”), mechanism of the interaction (e.g., binding, cleavage, phosphorylation, microRNA regulation etc.), and directionality.

However, the resulting network does not always accurately reflect the cellular events occurring in the condition from which the high-throughput experiment is taken. The interactions stored in Metacore are known to exist in a specific set of conditions in certain organisms - not necessarily under the users experimental conditions. Most programs provide an option to filter the network interactions by particular disease, tissue or species, but this limits the ability to discover new potential interactions not previously associated with the user’s disease/tissue/species, which are revealed in the user’s experiment.

On the other hand, if filtering is not used, the resulting network will contain many false positives, i.e., interactions not truly present in the user’s experimental conditions. Furthermore, the input gene list used to generate the network is simply a list, and does not encapsulate all the information in the researcher’s expression data, such as time series variation. In summary, it is likely that only a subset of interactions produced in the generated network are active - forming an active subnetwork of the interaction network.

### Active subnetwork problem description and background

Ideker *et al.*[1] introduced a framework for identification of active subnetworks from an original network. This framework entails a problem that searches for connected regions of a molecular interaction network that show significant changes in expression over a particular subset of the conditions.

Many researchers have since investigated this problem. Ultimately, the methods developed to solve this problem involve two parts: 

• the scoring function: how do you score a connected region of genes and interactions that reflects the likelihood that the region is active?

• the search method: how do you search among the connected regions for the regions with the highest scoring function (a global optimization problem)?

In Ideker’s work, the method proposed uses the statistical scores of individual genes obtained from gene expression data to derive an overall statistical score for every candidate subnetwork and uses simulated annealing to search from high-scoring subnetworks.

Ideker *et al.* showed that the second step in the problem, that is, searching for a connected subnetwork with the highest score is an NP-hard problem. Since then, various efforts have attempted to use heuristics to address the problem, including simulated annealing [1,2], local greedy search [3-6] and mathematical programming based methods [7,8].

Ideker’s method treats each gene’s contribution to the scoring function as fixed, because its differential expression (DE) status is determined only once. An alternative approach to active subnetwork searching is to adjust the classification of whether a gene is DE or not, according to the classification of its neighbors in an active subnetwork. By looking at the local neighborhood of a gene, and the expression levels of these genes, we can adjust what we consider to be true differential expression. By using an iterative approach, one can find an active subnetwork based on the genes adjusted DE classification.

Wei and Li [9] adopt such an approach. They use an iterative algorithm to assign a binary value to each gene in the original network that reflects the true DE state of the gene. Genes assigned to a true DE state belong to the active subnetwork, while the remainder of the genes do not belong to this subnetwork. In each iteration, a gene is assigned to a state by comparing the posterior probability of each state assignment (true DE or not). The posterior probability of a state is the product of the likelihood of observing the expression data given the state (a “noise model” - see later), and a prior probability of the state. A Markov Random Field (MRF) [10] is used to create the prior probabilities by exploiting dependencies between state assignments of neighboring genes in the network. Global parameters used in the noise model and MRF are updated at each iteration based on current state assignments using an iterated conditional mode algorithm [11].

Some methods have attempted to use observations on edges in their scoring function [2]. The advantage of edge-based methods is that returning a list of proteins (nodes) does not tell you anything about which interactions are active in the condition. Node-based methods rely on connecting all interactions in the original network that exist between the active nodes. In fact, nodes can be connected through multiple alternate interactions, and through indirect paths. It is useful to know exactly which interactions are active in the condition, and responsible for carrying out the biological function of interest.

One measure of edge strength is the correlation of gene expression between the two adjacent nodes [12,13]. Qiu *et al.* developed a tool RegMOD which classifies genes as “active” or “not active” using a support vector regression method where a diffusion kernel matrix specifies the relationship between adjacent genes using the Pearson correlation between gene’s expression levels [14].

Ma *et al.* claim to be the first to use both node and edge-based measures in the scoring function [15]. In particular, they use the F-statistic to measure gene differential expression, and an expected conditional F statistic (ECF) to measure gene-gene differential correlation across multiple observations between groups.

In a related study, Jaimovich *et al.*[16] predict ground truth state of protein-protein interactions (PPIs) from large PPI databases. They use protein interaction and cellular location assay data as noisy observation data of the ground truth state of each interaction (present or not). A relational markov network is used to create dependencies between neighboring interactions.

#### Function annotation in protein-protein interaction networks: A parallel problem domain

We now describe another problem domain in the area of biological network characterization which has arisen in parallel to the active subnetwork problem. Researchers working in each problem domain have generated tools and algorithms used by researchers in the other domain. This other problem domain concerns the annotation of previously unannotated proteins in protein-protein interaction (PPI) networks. Problems in this domain focus on optimizing the assignment of function labels to proteins with unknown function in a PPI. Solutions to this problem can also involve use of a scoring function that scores the likelihood of assigning a label to a given protein, and a search method to find the optimal assignment over all proteins in the network.

Deng *et al.* use a scoring function which employs a Markov Random Field that creates a dependency between neighboring proteins. The likelihood of protein being given a particular label assignment (e.g., label “A”) depends on the number of proteins assigned to that label in a local neighborhood in the PPI [17]. Letovsky and Kasif developed a protein labeling algorithm for a partially unlabeled PPI network by using a binomial model of the probability based on the local neighborhood, and a Markov Random Field belief propagation algorithm [18].

In an improvement to the work of Deng *et al.*, Lee *et al.* (the same team of researchers) modified the scoring function and used a multivariate logistic regression approach. In the new function, the likelihood of assigning a given protein to a label (e.g., label “A”) depends on multiple factors, where a factor is created for each label in the set of possible protein function labels. The factor reflects the number of proteins in the local neighborhood assigned to the particular label (e.g., label “i”), weighted by a unique parameter for the two labels (e.g., a parameter for label “A” with label “i”) [19].

#### Comparison of methods in two problem domains

We have described how MRFs have been used in the protein function identification problem domain [17,19], as well as in the active subnetwork problem domain [9,20] to reflect relationships between neighboring genes and proteins in biological networks. Lee’s modification of the protein label assignment MRF model to incorporate multiple factors that reflect the varying influence of neighboring protein label assignments according to the two labels in question, has not yet been extended to the active subnetwork problem. There is a good biological motivation in the active subnetwork problem space to exploit such multivariate techniques, as we describe shortly.

### A novel approach to discovering functional active subnetworks with a mechanism-dependent scoring function

#### Method summary

Our work builds on the above literature to learn active subnetworks, where we specify that the probability that an interaction is assigned to the “active” state depends on the state of each neighbor interaction and and where the level of dependence varies according to the mechanism assigned to the two interactions. This work can be seen therefore as a modification of the work of Wei and Li to incorporate some of the advances made by Lee *et al.*, with respect to weighting each neighbor’s influence according to pre-defined label assignments, rather than treating each neighbor as equally influential.

We use the controlled vocabulary provided by GeneGO Inc. to specify the mechanism labels in our problem. Table 1 states the symbols used in Metacore and this paper for the interaction mechanisms in Metacore.

**Table 1:** Metacore mechanisms and symbols

**Mechanism** **symbol**	**Description**
B	Binding - compound binds the enzyme or receptor
C	Cleavage
CM	Covalent Modification
+P	Phosphorylation
-P	Dephosphorylation
T	Transformation
Tn	Transport
Z	Catalysis
TR	Transcription regulation
M	MicroRNA binding

Our program written in the R language [21] reads in a network of interactions from Cytoscape [22] generated SIF file, as well as the Cytoscape annotation files which specify the interaction mechanism for each interaction in the SIF file. Our program also reads in a vector of correlation coefficients, considered to be a noisy observation of the true state of the interaction in that experiment, or can alternatively calculate the correlation vector from an input gene expression matrix. The algorithm assigns states (active or non-active) to the interactions in the network. The algorithm uses a probabilistic graphical model which incorporates both a noise model and a MRF over the interactions in the network to learn a final system state, which is described in section “A probabilistic graphical model framework”.

In this manuscript, we have used networks generated by Metacore from a list of differentially expressed genes. The networks are imported into Cytoscape using the plugin CytoscapeNetPlugin.jar, where the SIF files are exported. However, any Cytoscape network with edge labels containing mechanism annotations can be read into R and used in our algorithm.

The MRF uses parameters to express the dependence of an interaction’s state assignment on its neighbor’s state. While Wei and Li use a single parameter to express this dependence (the “single parameter approach”) [9], we learn the value of multiple parameters, where a parameter exists for each unique pair of mechanisms. The parameter expresses the relationship between a pair of neighboring interactions annotated with this pair. With a larger set of parameters, a more complex function is used to model the dependence of each interaction on a local neighborhood in the network. We iteratively learn this larger set of parameters and infer the state of interactions, to converge to an active subnetwork. We call this approach a “full parameter” approach.

We compare this approach to the single parameter approach and Ideker’s JActiveModules using artificially created original networks with simulated data. We then apply the single and full parameter approaches to learn active subnetworks from real expression data, and discuss how the full parameter approach highlights different functional aspects of the active subnetwork for closer consideration by biological scientists.

We now justify why we believe it is appropriate to split up the parameter to weight a neighbor’s influence according to its mechanism in the active subnetwork problem domain.

#### Assumptions about mechanism-mechanism pairs in active subnetworks

In this work, we assume that interaction mechanisms are correlated with other interactions mechanisms in active subnetworks, which we refer to as mechanism-mechanism (M-M) correlation here, and define more formally later. The level of correlation varies according to the two mechanisms involved.

To justify this assumption, we first consider hypothetical regions of a network made up of a subset of mechanisms, using evidence and citations of such regions in real biological networks. 

1. A phosphorylation pathway that contains a series or “cascade” of phosphorylation and de-phosphorylation interactions. In such a cascade, interactions with mechanisms Phosphorylation (+P) or De-phosphorylation (-P) are typically physically located next to or close to one another. We would therefore expect there to be a high mechanism-mechanism correlation for the mechanism pairs {+*P*,+*P*},{−*P*,−*P*}, and potentially {+*P*,−*P*}. That is, we assume if part of the signaling cascade is active in a given condition, it is likely that the remaining interactions in the cascade are also active in the condition. As an example, the ERK1/2 cascade is highly conserved, and depending on the input stimulus and cell type, regulates various cellular processes such as proliferation, differentiation, and cell cycle progression [23]. In order for the cellular processes to be properly carried out, all phosphorylation steps in the pathway must be carried out; i.e., in order for ERK 1/2 to relocate to the nucleus and phosphorylate its nuclear targets, all previous phosphorylation steps must have occurred.

2. A large protein complex made up of multiple protein subunits that bind together through a binding mechanism (B). The formation of proteins from multiple subunits is prolific throughout biology, and genes corresponding to protein complex subunits are often coexpressed in a condition [13]. As one example, fibrin is glycoprotein made up of three pairs of non-identical polypeptide chains: which are the fibrinogen alpha, beta and gamma subunits. Here, we expect a large mechanism-mechanism correlation for the mechanism pair {*B*,*B*}.

3. A cleavage cascade that contains a series of cleavage (C) steps. For example, the blood coagulation process involves the activation of multiple coagulation factors, which through a cascade of protein cleavage events, converge on thrombin, which in turn cleaves fibrinogen to generate a network of cross-linked fibrin that is involved in blood coagulation [24]. We would expect a large mechanism-mechanism correlation for the mechanism pair {*C*,*C*}.

4. A transcription regulatory region in which a transcription factor (TF) regulates multiple targets via a transcription mechanism (TR). Typically, a particular TF becomes biologically activated (e.g., through a mutation or phosphorylation event), locates to the nucleus, and is sufficiently expressed to regulate multiple potential gene targets. This would reflect the well documented 1-many behavior of TF bindings in biological conditions. See for example Yu *et al.*[25] who showed that target genes of a regulator tend to be co-expressed, and that the level of co-expression is higher when multiple transcription factors are involved. We also know that transcription regulatory cascades appear in transcription regulatory networks [26]. We therefore expect a high correlation for the {*TR*,*TR*} mechanism pair in an active subnetwork with transcription regulatory regions.

5. A transcription region involving microRNA (miRNA) activity. miRNAs are a 22-23nt RNA transcript which are known to target mRNA and either break down the transcript, or prevent its translation to a protein [27,28]. The mechanism annotation is (M). It has been shown that miRNAs often play a role in controlling mRNA transcript levels by participating in simple and complex circuits [29] such as single-input motifs (SIMs) (one miRNA to multiple targets) and multiple-input motifs (MIMs) (multiple miRNAs bind to multiple targets to provide a coordinated regulation of targets) [30,31]. Because miRNAs participate in SIMs and MIMs we expect high correlation for the pair {*M*,*M*} in an active subnetwork where miRNAs are functional.

6. A complex regulatory circuit made up of miRNAs and TF activity. For example, it has been shown that miRNAs can bind to the gene target of a TF, or to the TF itself, in a feed-forward loop [32,33]. In such a region interactions with TR mechanisms neighbor interactions with M binding mechanisms, and we expect an active subnetwork containing such a region to have high a correlation for the pair {*TR*,*M*}.

We seek to find active subnetworks by using the assumption that true biological networks are likely to contain regions that are largely, (though not entirely), made up of interactions belonging to a subset of mechanisms. The mechanisms group together to carry out a meaningful biological function within the region. We have justified this assumption in this section, by providing multiple examples with evidence of such mechanism groupings in several canonical GeneGO Pathway Maps.

#### M-M pair rich active subnetworks

We predict our method will have the greatest predictive accuracy in recovering active subnetworks which meet certain properties. First, we provide some definitions. We define an M-M pair as a set of two mechanisms in **IM**. If n= |*IM*|, there are $\frac{n\times (n+1)}{2}$ possible M-M pairs allowing pairs of the same mechanism. First, we divide M-M pairs into two types: *correlated M-M pairs* and *non-correlated M-M pairs*. We now define an interaction-interaction pair (I-I pair) as a pair of neighboring interactions in the network. Consider one of the possible M-M pairs, which we call M1-M2 for convenience. We say an I-I pair is labeled M1-M2 if one interaction from the pair is labeled M1 (i.e., has an annotated mechanism M1), and the other interaction is labeled M2 (i.e., has an assigned mechanism M2). Further, each interaction in an I-I pair has a true activity state in the original network: active or non-active. (It is this state that we try to recover in the active subnetwork recovery problem.)

We now describe the properties of the original network that will generate good results with the full parameter approach. Each pair M1-M2 in the set of *correlated M-M pairs* should meet the following criteria. Consider only I-I pairs labeled M1-M2 in the network. This set of interactions should be enriched for I-I pairs where the two interactions are in the same activity state. This means, among I-I pairs labeled M1-M2, the number of pairs where both interactions in the pair are active, or both interactions are not active, should occur more frequently than expected by random assignment of activity state to interactions. Specifically, the network should have a greater number of I-I pairs labeled M1-M2 that are in the *same* state *relative to* the number I-I pairs labeled M1-M2 in *different* states (i.e. one interaction is active, and the other not active). Such a finding suggests I-I pairs labeled M1-M2 are co-activated; that is, when one interaction labeled M1 is active in a condition, its neighbor labeled M2 is also likely to be active. These pairs will have a high mechanism-mechanism correlation.

For a pair M1-M2 in the set of *non-correlated pairs*, the network should have a similar number of I-I pairs labeled M1-M2 in *different* states relative to the number of I-I pairs labeled M1-M2 in the *same* state. I-I pairs labeled M1-M2 in this scenario are *not* co-activated in the condition, and are not expected to have a high mechanism-mechanism correlation.

If the original network has these properties, we describe the active subnetwork as an “M-M pair rich active subnetwork”. Such a network produces a range of mechanism-mechanism correlations, allowing the full parameter method to fine-tune the dependencies of interaction activity states on a neighbor’s activity state, according to the mechanisms assigned to the interactions.

**Figure 1:**
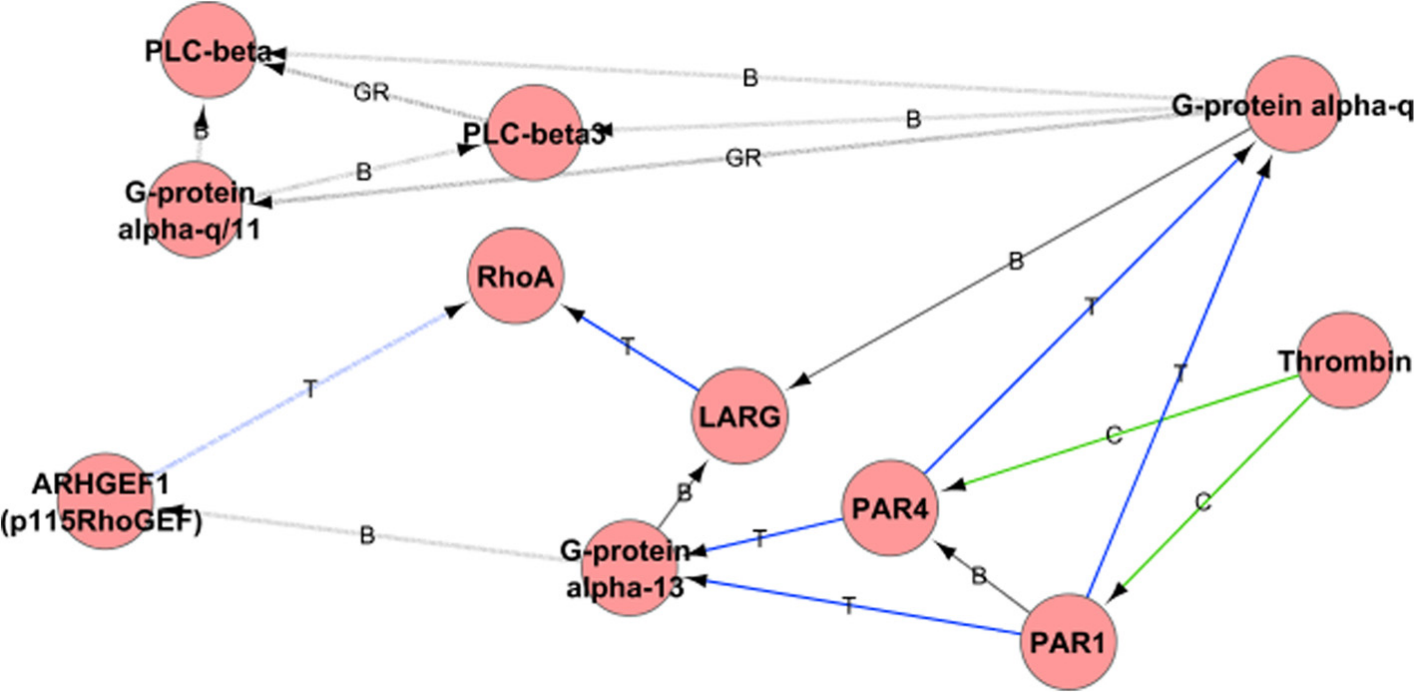
**Part of blood coagulation pathway downloaded from GeneGO Inc.** Interactions are colored by mechanism; grey for binding (B), green for cleavage (C) and blue for transformation (TR). Solid lines indicate active interactions (the active subnetwork), while dotted lines indicate the remaining non-active interactions. The figure highlights a region of the active subnetwork rich in interactions of pairs {*C*,*C*},{*C*,*T*} and {*T*,*T*}. In the active subnetwork, Thrombin cleaves two protein receptors (PAR1 and PAR4). These receptors then transform G-protein alpha-13, enabling it to effect the blood coagulation process further. On the contrary, there is no strong correlation for the pair {*B*,*T*}, as about the same number of I-I pairs with this label are in the same state (e.g., G-protein alpha-q (B) LARG (active), LARG (T) RhoA (active)), as in a different state (e.g., PAR4 (T) G-protein alpha-13 (active),G-protein alpha-13 (B) ARHGEf1 (not active)).

When viewing only the active or non-active part of the original network (the active subnetwork), it should appear to contain regions with many I-I pairs labeled with M1-M2, if M1-M2 is a *correlated M-M pair*. Figure 1 show an example of an M-M pair rich active subnetwork in a Blood Coagulation Pathway Map downloaded from GeneGO Inc.

In our solution, we will see that mechanism-mechanism correlations for each M-M pair are not known and are learned from the data in the form of parameters. Having provided the background and justification for our approach, we now introduce our solution to the active subnetwork problem.

## Methods

### A probabilistic graphical model framework

We use a probabilistic graphical model framework to address our problem of finding active subnetworks within networks generated with GeneGO Metacore tool from an uploaded experimental dataset. The probabilistic graphical model contains two components. The first component describes the likelihood of observing the correlation of expression profiles between edges in the network given the state (active or non-active) of all the interactions in the network (the Noise Model). The second component creates dependencies between the states of neighboring interactions in the physical network (the MRF component).

### Random variables, parameters and assumptions

#### Notation

We use the following notation. We use capital letters to denote RVs (eg *X*) and lower case letters denote a particular realization (or observation) of a RV (e.g., *x*). Subscripted letters denote a particular RV from a class of RVs (e.g., *X*_1_). **Bold font** denotes a vector of RVs belonging to the same class (e.g., **X**={*X*_1_,*X*_2_…*X*_*n*_}). A horizontal bar over a value denotes a RV is not equal to this value (e.g., *x* =$\bar{j}$ denotes x is not equal to j). For a set of variables, a horizontal bar over a RV denotes all variables in the set except this RV (e.g., for the set S, $S_{\bar{i}}$) implies all RVs in S except i.The size of a vector (or set) is denoted by || (e.g., |*X*|).

#### Network and graphical model definitions

We define the original network as a graph *G*=(*V*,*I*) with a set of components *v*_*i*_∈**V** and a set of interactions *I*_*i*,*j*_∈**I** between components *v*_*i*_ and *v*_*j*_. **Y** = (*Y*_1_,*Y*_2_…*Y*_*p*_) is a vector of RVs comprising the time-course gene expression profiles for *p* genes in the original network. Each **Y**_*i*_∈**Y** is itself a vector of size *n*∗*t* of observations for each node *v*_*i*_ in the network with *n* replicates taken over *t* time points during the time course. Each interaction *I*_*i*,*j*_ is pre-assigned by Metacore to an interaction mechanism, which we denote by IM(*I*_*u*,*v*_), where IM(*I*_*u*,*v*_) ∈{*B*,*C*,*CM*,+*P*,−*P*,*T*,*Tn*,*Z*,*Tr*,*M*}.

To construct the graphical model, we convert interactions in *I*^*P*^ to random variables. In the graphical model, there are several classes of random variables. **Q** = ($Q_{1,1},\ldots Q_{1,n_{1}},Q_{2,1}\ldots Q_{2,n_{2}}\ldots Q_{g,n_{g}})$ is a vector of latent discrete RVs, where *Q*_*u*,*v*_ is the RV corresponding to interaction *I*_*u*,*v*_ in the original network, and *n*_*i*_ is the final neighbor of source node *v*_*i*_. We refer to *IM*(*Q*_*u*,*v*_) to specify the interaction mechanism of the random variable *Q*_*u*,*v*_ in the graphical model.

Each entry *Q*_*u*,*v*_ in **Q** specifies a probability distribution over each state in the state space. Each *Q*_*u*,*v*_ corresponds to a state assignment (or realization) of *Q*_*u*,*v*_. **Q** thus specifies the full system realization for all the interactions in the putative network. The state space of *Q*_*u*,*v*_ may be large as we shall see, however, the distribution for *Q*_*u*,*v*_ only specifies non-zero probabilities for two states, the active or non-active state. We use *A*(*Q*_*u*,*v*_)=1 to denote the event that *Q*_*u*,*v*_ is in the active state and *A*(*Q*_*u*,*v*_)=0 to denote the event that *Q*_*u*,*v*_ is in the non-active state.

**R** comprises the set of sample correlation statistics calculated from the expression data **Y**. *R*_*u*,*v*_ is an observed RV in the graphical model and corresponds to the Spearman correlation between expression variables (*Y*_*u*_ and *Y*_*v*_) over a single time-course experiment.

#### Model parameters

The parameters in this model can be thought of as latent variables. The parameters include: 

• *Θ* - A distributional parameter used to determine the likelihood of values in **R** given the current system realization.

• *Ψ* - A set of parameters which describe the relationship between adjacent RVs in the MRF component of the graphical model which creates a dependency between the state of neighboring interactions in the putative network.

#### Model assumptions

We create the following model assumptions: 

• Each sample correlation *R*_*u*,*v*_ between RVs *Y*_*u*_ and *Y*_*v*_ is calculated using the Spearman correlation coefficient and thus there are no assumptions on the distribution of *Y*_*u*_ or *Y*_*v*_.

• Each RV *Q*_*u*,*v*_ is coupled to other RVs in **Q** via the MRF *C*_*Q*_. The parameters of the MRF are learned and specified in *Ψ*.

### Graphical model representation

We define the graphical model formally as a probability distribution *μ* which factorizes over a graph. The nodes in the graphical model comprise the RVs defined in “Network and graphical model definitions” corresponding to interactions in the original network and parameters defined in section “Model parameters”. The edges of the graphical model describe conditional independence properties between the RVs in accordance with graphical model theory.

**Figure 2:**
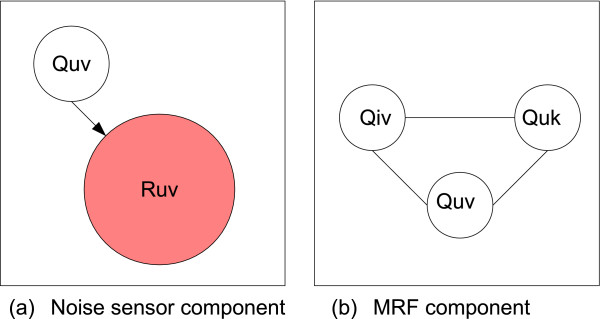
**The two components of the graphical model comprise (a) a noise model, and (b) a Markov Random Field (MRF).** The graph contains a directed component (noisy sensor) and an undirected component which represents the MRF. The figure shows cliques in the graphical model for an example RV *Q*_*u*,*v*_. The noise model specifies that the observed RV *R*_*u*,*v*_ is independent of all other RVs except its parent *Q*_*u*,*v*_. The MRF states that the marginal likelihood of *Q*_*u*,*v*_ is independent of all other RVs in the graphical model except *Q*_*i*,*v*_ and *Q*_*u*,*k*_. Each interaction RV in **Q** participates in a noise model clique, and multiple cliques in the MRF.

Figure 2 contains a diagram of two different types of cliques within the graphical model.

The noise model specifies the conditional likelihood that RV *R*_*u*,*v*_ is equal to its observed value *R*_*u*,*v*_ given the state of parent variable *Q*_*u*,*v*_:

(1) $$l_{u,v}=\mu (R_{u,v}=r_{u,v}|q_{u,v})$$

The MRF component creates a dependency between *Q*_*u*,*v*_ and a local neighborhood of interactions denoted by *∂*_*u*,*v*_. The MRF specifies the conditional probability of state assignment *Q*_*u*,*v*_ given a potential function *ψ*_*u*,*v*_. The potential function *ψ*_*u*,*v*_ is a function of the state realization of the local neighborhood denoted by $q_{\partial_{u,v}}$ and the parameter set *Ψ*.

(2) $$\mu (Q_{u,v}=q_{u,v})\propto \;\exp\;(\psi_{u,v}(q_{u,v},q_{\mathrm{\partial u},v},\Psi ))$$

We describe the joint likelihood function when we describe the learning and inference algorithms.

### Noise model

We now define the conditional likelihood function used to express the clique potential *Ψ* for cliques in the noise model. We provide an explicit formula for the conditional likelihood term in equation 1.

The conditional likelihood term reflects the likelihood of observing the correlation data **R** given the system state. For noise model clique for example RV *Q*_*u*,*v*_, this is a statistical test on RV *R*_*u*,*v*_. We consider the two alternative hypotheses on *Q*_*u*,*v*_ for *k*∈{0,1}, corresponding to whether the RV is assigned to the active state (k=1) or non-active state (k=0). Assigning the interaction to a state, is like assigning the interaction to a class. Each class k consists of a population of interactions. The population can be described by a correlation coefficient population parameter *ρ*_*k*_, which expresses the mean level of correlation between adjacent genes, for interactions in class k.

The null hypothesis is: *H*_0_:*A*(*Q*_*u*,*v*_)=0; i.e., *I*_*u*,*v*_ is not active in the experimental condition, *A*(*Q*_*u*,*v*_) is in class k=0. For interactions in class k=0, we assume *R*_*u*,*v*_ is sampled from a population with parameter *ρ*_*k*=0_ =0. That is, for interactions in this population, there is, on average, no correlation of expression between two genes adjacent to the interaction.

The alternative hypothesis *H*_1_ is: *A*(*Q*_*u*,*v*_)=1, i.e., the interaction between nodes u and v is active in the experimental condition. For interactions in class k=1, we assume the correlation is centered on some number *ρ*_*k*=1_>0 i.e., there is, on average, some correlation of expression between the two adjacent genes over the time-course. The assumption is if the genes tend to be co-activated at the same time points, it is likely that the interaction is active.

The likelihood function seeks to find the probability of obtaining the sample correlation coefficient, i.e., *R*_*u*,*v*_=*r*_*u*,*v*_, for each alternative hypothesis.

We use a Fisher transformation in R package **psych**[34] to generate a new RV *Z*_*u*,*v*_ from *R*_*u*,*v*_, where *Z*_*u*,*v*_ is a normally distributed RV.

The Fisher transformation is:

$$Z=\frac{1}{2}\;\log\;\frac{1+r}{1-r}=\text{artanh}(r)$$

*Z*_*u*,*v*_ is approximately normally distributed with mean that is Fisher transform of population CC *ρ*_*k*_ for *k*∈{0,1}, and standard error $\sqrt{n-3}$ for sample size n.

$$Z∼N(\frac{1}{2}\;\log\;\frac{1+\rho_{k}}{1-\rho_{k}},\frac{1}{\sqrt{n-3}})$$

We first consider the case k=1. We do not know population parameter *ρ*_*k*=1_, however assume we have an estimate of this parameter *r*_*α*_, with corresponding Fisher transformation *z*_*α*_.

In our model, equation 1 becomes:

(3) $$\;l_{u,v}=\text{Pr}(Z_{u,v}<z_{u,v}|Z_{u,v}∼N(\mu =z_{\alpha },\text{sd}=\frac{1}{\sqrt{n-3}}))$$

The transformed likelihood function is a distribution function which has the property that when *R*_*u*,*v*_ = *r*_*α*_,*l*_*u*,*v*_=*Pr*(*Z*_*u*,*v*_<*z*_*α*_|*A*(*Q*_*u*,*v*_=1))=0.5. *l*_*u*,*v*_ increases as *R*_*u*,*v*_ exceeds *r*_*α*_, and decreases in the other direction. Therefore, the larger the value of *R*_*u*,*v*_ relative to *r*_*α*_, the greater the value of the likelihood term.

For *H*_0_ : *A*(*Q*_*u*,*v*_)=0, we convert *R*_*u*,*v*_ to a t statistic *t*_*u*,*v*_ and perform a one-tailed t test. Equation 1 becomes:

(4) $$l_{u,v}=\mu (T_{u,v}>t_{u,v}|A(Q_{u,v})=0)∼T(t_{u,v},\text{df}=n-2).$$

If *R*_*u*,*v*_ = 0, then *l*_*u*,*v*_=*μ*(*T*_*u*,*v*_>0|*A*(*Q*_*u*,*v*_=0))=0.5. *l*_*u*,*v*_ now decreases as *R*_*u*,*v*_ increases above 0. Therefore, the smaller the value of *R*_*u*,*v*_ relative to 0, the greater the value of the conditional likelihood term in calculating *μ*(*A*(*Q*_*u*,*v*_)=0).

These tests apply where we assume a positive correlation of expression for *H*_1_, i.e., *ρ*_1_>0. For negative correlation *r*_*u*,*v*_<0, we use the absolute value of the correlation coefficient |*z*_*u*,*v*_|.

Thus *Θ*={*r*_*α*_} and the parameter *r*_*α*_ is computed using a maximum likelihood estimator discussed in Section “M-step”.

### The MRF component

We apply MRF modeling tools developed in the field of Vision Analysis (see [35]) to this problem.

As stated in equation 2, we define a probability term for each *Q*_*u*,*v*_ as proportionate to exp(*ψ*_*u*,*v*_), where the potential function *ψ*_*u*,*v*_ depends only on the state of the local neighborhood of interactions *∂*_*u*,*v*_ denoted by *q*_*∂u*,*v*_.

The single parameter and full parameter approaches both define the local neighborhood *∂*_*u*,*v*_ as the set of interactions which are immediate neighbors in the physical network. Both approaches specify the potential function *Ψ* over cliques of size 1 and 2 in the graphical model. However, the single parameter and full parameter approach define *ψ*_*u*,*v*_ differently.

#### Potential function dependent on neighbor state only

We use the c-color coding distribution [11] that assigns random variables to one of a discrete set of colors of size c. The Ising model is a special case of the c-color coding distribution for c=2 colors, the active state *A*(*Q*_*u*,*v*_)=1 and the non-active state *A*(*Q*_*u*,*v*_)=0.

The Gibbs total energy function is defined over all pair-site cliques (cliques of size 1- and 2-), as

(5) $$P(q;\Psi )\propto \;\exp\;(\gamma_{0}n_{0}+\gamma_{1}n_{1}-\beta n_{0,1})$$

where *n*_0_ is the number of interactions with state 0, *n*_1_ is the number of interactions with state 1, and *n*_0,1_ is the number of interactions with different states, and *Ψ*=(*γ*_0_,*γ*_1_,*β*). There is a normalization constant omitted from this equation.

The conditional probability of a RV *Q*_*u*,*v*_ taking a given state *k*∈{0,1} is:

$$\mu (q_{u,v}=k|\Psi ,q_{\mathrm{\partial u},v})\propto \;\exp\;(\psi )$$

where

(6) $$\psi =\gamma_{k}-\beta \eta_{u,v}(\bar{k})$$

where $\eta_{u,v}(\bar{k})$ denotes the number of neighbors of *Q*_*u*,*v*_ having a state ≠ k.

*Ψ*={*γ*_0_,*γ*_1_,*β*} and the parameters in *Ψ* are computed with a maximum likelihood estimator discussed in section “M-step”.

#### Potential function as a fully parametric model dependent on neighbor state and mechanism

In the full parameter approach the potential function *Ψ* now depends on the state and mechanisms of the neighbors of *Q*_*u*,*v*_. Each interaction can be active or inactive, but will also be assigned a mechanism imported from Metacore which exists in the original network, denoted IM, i.e., *IM*={*B*,*C*,*CM*,+*P*,−*P*,*T*,*Tn*,*Z*,*Tr*,*M*}. Each interaction can therefore be in 2∗|*IM*| states. Lets call this new state space *IM*_*state*, e.g.,

$$\;\begin{array}{l} \text{IM}\text{\_}\text{state}=\;\{B_{0},C_{0},CM_{0},+P_{0},-P_{0},T_{0},Tn_{0},Z_{0},Tr_{0},M_{0},\\ \;B_{1},C_{1},CM_{1},+P_{1},-P_{1},T_{1},Tn_{1},Z_{1},Tr_{1},M_{1}\} \end{array}$$

The model now requires the c-color method for *c*>2.

We reduce the state space for each interaction as follows. We first introduce some terminology. For each state in the state space *k*∈*IM*_*state*, we can describe state “k” as having an interaction mechanism component denoted “IM(k)” and an activity level component denoted “A(k)”. Similarly, we described RV *Q*_*u*,*v*_ assigned to state k as having the same two components. For example, if *q*_*u*,*v*_=*B*_0_, it consists of *IM*(*Q*_*u*,*v*_)=*B* and *A*(*Q*_*u*,*v*_)=0. Metacore makes a hard assignment of a mechanism to each interaction *I*_*u*,*v*_ in the original network. This means that its corresponding RV *Q*_*u*,*v*_ in practice can only ever be in two states for *k*∈*IM*_*state*, which are the active and non-active states where the interaction mechanism component of k is the annotated value of that mechanism in the knowledge database; i.e., *IM*(*k*)=*IM*(*I*_*u*,*v*_),*A*(*k*)=1 and *IM*(*k*)=*IM*(*I*_*u*,*v*_),*A*(*k*)=0. In graphical model and probability language, this means that the conditional likelihood *μ*(*Q*_*u*,*v*_=*k*)=0 where *IM*(*k*)≠*IM*(*Q*_*u*,*v*_). For example, if *IM*(*Q*_*u*,*v*_)=*M* (a MicroRNA binding interaction), then *μ*(*Q*_*u*,*v*_=*M*_1_)∈(0,1) and *μ*(*Q*_*u*,*v*_=*M*_0_)∈(0,1), and *μ*(*Q*_*u*,*v*_=*B*_0_)=*μ*(*Q*_*u*,*v*_=*B*_1_)=*μ*(*Q*_*u*,*v*_=+*P*_0_)…=0. This reduces the non-zero state space for each interaction and therefore the number of conditional likelihood terms we must calculate for the RVs in **Q**.

Under the full parameter model, we have parameters *γ*_*k*_ for each state k in *IM*_*state* and parameters *β*_*k*,*l*_ for each pair of states k and *l*∈*IM*_*state*. This would give a total energy function:

(7) $$\;\begin{array}{l} \mu (q;\Psi )\propto \;\exp\;(\underset{1\leq k\leq |\text{IM}\text{\_}\text{state}|}{\sum }\gamma_{k}n_{k}-\sum \underset{1\leq k\leq l\leq |\text{IM}\text{\_}\text{state}|}{\sum }\beta_{k,l}n_{k,l}) \end{array}$$

where *n*_*k*_ is the number of interactions in state k, and *n*_*k*,*l*_ is the number of distinct neighbor pairs colored (*k*,*l*).

The conditional likelihood function is:

$$\mu (Q_{u,v}=k|\Psi ,q_{\mathrm{\partial u},v})\propto \;\exp\;(\psi )$$

where

(8) $$\psi =\gamma_{k}-\underset{l\in \text{IM}\text{\_}\text{state}}{\sum }\beta_{k,l}\eta_{u,v}(l)$$

where *β*_*k*,*l*_=*β*_*l*,*k*_ and *η*_*u*,*v*_(*l*) is the number of neighbors of *Q*_*u*,*v*_ having state l.

Many of the networks we are examining are smaller than 1000 nodes, and the original network is fairly sparse (small number of edges). Learning parameters from such small training data could lead to over-fitting the parameters, so we reduce the number of parameters by setting *β*_*k*,*l*_ to zero where k and l have different activity state components, i.e., *A*(*k*)≠*A*(*l*). The remaining non-zero *β* terms therefore include only terms where states k and l possess the same activity state components.

In the reduced parameter format, we say $\Psi =\{\gamma_{s_{a}},\beta_{s,t}\}$ for *a*∈{0,1},*s*∈*IM*,*t*∈*IM* and equation 8 becomes:

(9) $$\psi =\gamma_{s_{a}}-\underset{t\in \text{IM}}{\sum }\beta_{s,t}\eta_{u,v}(t_{ā})$$

where $\eta_{u,v}(t_{ā})$ is the number of neighbors of node *Q*_*u*,*v*_ with mechanism t not in state a.

The parameters in *Ψ* are computed with a maximum likelihood estimator discussed in section “M-step”.

### EM inference and learning

We implemented the inference and learning algorithm using the expectation-maximization (“EM”) algorithm to find the value of latent variables in our model [36,37]. The EM algorithm has the advantage that each step of the algorithm increases the complete data log likelihood function (see [38]). In the work of Wei and Li [9], the inference step in the EM algorithm is performed with iterative conditional mode (ICM) algorithm [39].

In the EM algorithm, we try to find the state of latent variables *A*(*Q*_*u*,*v*_)∈{0,1} which maximizes the expected value of the complete data log likelihood function.

The expected complete data log likelihood function combines the noise model with the MRF component as follows:

(10) $$\begin{array}{l} F=E(\;\log\;\mu (A(Q),R|\Psi ,\Theta )|R,\hat{\Psi },\hat{\Theta })\\ \;=\;\log\;\underset{(u,v)\in Q}{\prod }\underset{j\in \{0,1\}}{\prod }\mu (R,A(Q_{u,v})=j|\Theta ,\Psi )\\ \;=\underset{(u,v)\in Q}{\sum }\underset{j\in \{0,1\}}{\sum }[\log(\mu (R|A(Q_{u,v})=j,\Theta ))\\ \;+\;\log\;(\frac{\mu (A(Q_{u,v})=j|Q_{\partial_{u,v}},\Psi )}{\underset{k}{\sum }\mu (A(Q_{u,v})=k|Q_{\partial_{u,v}},\Psi )})]\\ \;\times \;\mu (A(Q_{u,v})=j|R,\hat{\Psi },\hat{\Theta }) \end{array}$$

where $q_{\partial_{u,v}}$ is a vector of RVs, where each RV corresponds to the state of an interaction in the set of neighbours *∂*_*u*,*v*_ i.e., $q_{\partial_{u,v}}$ is a vector over all the possible states of the neighbors of *Q*_*u*,*v*_, current estimates of {*Θ*,*Ψ*} denoted as $\left\{\hat{\Theta },\hat{\Psi }\right\}$, and $\mu (A(Q_{u,v})=j|R,\hat{\Psi },\hat{\Theta })$ is the posterior marginal probability of *A*(*Q*_*u*,*v*_) calculated with previous parameter estimates.

The EM algorithm iterates calculating the posterior marginal distributions with maximizing the complete data log likelihood function to find the values of parameters {*Θ*,*Ψ*} which optimize the function.

#### Expected value

In the inference step (the E-step), we calculate the posterior distribution of the latent variables given by $\mu (A(Q_{u,v})|R,\hat{\Theta },\hat{\Psi })$.

With only a single parameter, calculation of the posterior distribution for latent variable *Q*_*u*,*v*_ becomes (considering only the MRF component):

(11) $$\;\begin{array}{l} \mu (A(Q_{u,v})=j|Q_{\partial_{u,v}},\hat{\Psi })\\ \;=\underset{q_{\partial_{u,v}}\in Q_{\partial_{u,v}}}{\sum }\;\exp\;(\hat{\gamma_{j}}-\hat{\beta }\times |q_{\partial_{u,v}}=\bar{j}|)\times \mu (A(q_{\partial_{u,v}})) \end{array}$$

where $q_{\partial_{u,v}}$ is a possible realization of RV vector $q_{\partial_{u,v}}$, and $\left|q_{\partial_{u,v}}=\bar{j}\right|$ is the number of interactions not in state *j* in the set realization $q_{\partial_{u,v}}$.

To simplify the calculation, we use a heuristic modification of belief propagation adopted by Letovsky and Kasif [18]. Instead of summing over each possible neighbour realization, we calculate the expected number of neighbors in state $\bar{j}$ by using current values of the posterior distribution *μ*(*A*(**Q**)). These values are used to calculate a new posterior calculation for *μ*(*A*(*Q*_*u*,*v*_)).

(12) $$\begin{array}{lcrr} \mu (A(Q_{u,v})=j|Q_{\partial_{u,v}},\hat{\Psi })=\;\exp\;(\hat{\gamma_{j}}-\hat{\beta }\times E_{{\bar{j}}_{u,v}}) & & & \end{array}$$

where

(13) $$\begin{array}{lcrr} E_{{\bar{j}}_{u,v}}=\underset{i\in \partial_{u,v}}{\sum }\mu (A(Q(i))=\bar{j}) & & & \end{array}$$

i.e.,, $E_{{\bar{j}}_{u,v}}$ is the expected number of neighboring interactions in active state $\bar{j}$ based on current posterior distribution.

Calculation of the posterior distribution for the full parameter model modifies equation 12 so that *γ*, *β* and $E_{{\bar{j}}_{u,v}}$ become vectors, and the energy function appears as in equation 9.

#### M-step

In the M step we estimate parameters {*Ψ*,*Θ*} by maximizing the expected complete data log likelihood function in equation 10.

To find the maximum likelihood estimate (MLE) for *Θ*={*r*_*α*_}, we set the partial derivative of the complete data log likelihood function to zero, i.e., F= $\frac{\mathrm{\partial E}(\;\text{log}\;(A(Q),R|\Psi ,\Theta )}{\partial_{\Theta }}=0$, and the *Ψ* term cancels out. Thus the expected log likelihood term becomes for *Θ*:

(14) $$\begin{array}{l} ML_{\Theta }=\underset{(u,v)\in Q}{\sum }\underset{j\in \{0,1\}}{\sum }\;\log\;(\mu (R|A(Q_{u,v})=j,\Theta ))\\ \;*\mu (A(Q_{u,v})=j|R,\hat{\Psi },\hat{\Theta }) \end{array}$$

Given *Θ* contains *r*_*α*_ which only appears in the maximum likelihood term when j=1, we can set maximum likelihood for *Θ*=*r*_*α*_.

(15) $$\begin{array}{llll} ML_{\Theta } & = & \underset{(u,v)\in Q}{\sum }\log(\mu (Z_{u,v}=z_{u,v}|N(z_{\alpha },\frac{1}{\sqrt{n-3}})) & \\ & & \times \mu (A(Q_{u,v})=1|R,\hat{\Psi },\hat{\Theta }) \end{array}$$

Another option is to rank the probability (rank $\mu (A(Q_{u,v})=1|R,\hat{\Psi },\hat{\Theta })$) to weight the likelihood function. This can provide a more stable estimate when many RVs have large probabilities, by providing the largest weight to those *Z*_*u*,*v*_ values with the highest ranked probabilities.

To find the max likelihood assignment of *Ψ*, taking the partial derivative with respect to *Ψ*, the *Θ* terms cancel out. Maximum likelihood estimation of the parameters in *Ψ* must take into account the partition function. Solving maximum likelihood becomes intractable, and thus we use we use pseudo-likelihood (PL) approach[40]. The pseudo likelihood term is:

(16) $$\begin{array}{llll} PL_{\Psi } & = & \underset{(u,v)\in Q}{\sum }\underset{j\in \{0,1\}}{\sum }\log(\frac{\mu (A(Q_{u,v})=j|E_{{\bar{j}}_{u,v}},\Psi )}{\underset{k}{\sum }\mu (A(Q_{u,v})=k|E_{{\bar{k}}_{u,v}},\Psi )}) & \\ & & \times \mu (A(Q_{u,v})=j|R,\hat{\Psi },\hat{\Theta }) & \end{array}$$

where $\mu (A(Q_{u,v})=j|E_{{\bar{j}}_{u,v}},\Psi )$ is set out in equation 12.

The learning and inference algorithm is as follows, where the while loop is iterated until convergence of interaction state assignment is reached.

##### Algorithm 1 EM ALGORITHM FOR ACTIVE SUBNETWORK RECOVERY WITH HEURISTIC E-STEP (R)

## Results and discussion

### Validation using simulated correlation data for GeneGO pathway maps

To validate the performance of our model, we used known pathways downloaded as GeneGO Pathway Maps, from which we create active interactions as described below. Our aim is to recover the set of interactions in the original active subnetwork(s), starting with the original network and simulated correlation data for simulations *k*∈*N*. We term the set of interactions in the original active subnetwork **I**^∗^. We use four different algorithms to recover a set of active interactions for a simulation *k***A**_*k*_; **A**_*k*_ ⊆**I**. We evaluate the success of each of the four algorithms in comparing **A**_*k*_ to **I**^∗^.

The algorithms evaluated are the single parameter model (see section “Potential function dependent on neighbor state only”), the full parameter model (see section “Potential function as a fully parametric model dependent on neighbor state and mechanism”), JActiveModules [1] and a “maximum likelihood approach”. JActiveModules Cytoscape plugin is popular, widely used and often used for comparison purposes by other active subnetwork researchers (e.g., see [14]);. The maximum likelihood approach uses only the maximum likelihood configuration of states from optimizing the likelihood function from the noise model specified in equation 3. This method uses no prior information from a MRF.

#### Generation of original active subnetworks

To generate the active subnetworks, we recreate a GeneGO Pathway Map canonical Pathway Map using the Build Networks tool on the map Network Objects in Metacore with the Direct Interactions algorithm. This contains the “pathway interactions”. We then expand the pathway network with Metacore tools to add other “expanded interactions”, to form the final original network *G*.

We then generate the true active subnetwork within the original network as follows. We first describe the characteristics of network where we expect large performance gains to be observed using the full parameter approach over the single parameter approach.

Formally, we require that: 

1. for *some* mechanism-mechanism pairs (e.g., {*M*,*TR*}, interactions of the first mechanism have on average, of their neighbors of the second mechanism, a greater number are in the *same* activity state as the subject interaction than in a *different* activity state, and vice-versa (“correlated pairs”); and

2. for *other* mechanism- mechanism pairs (e.g., {*M*,*B*}, interactions of the first mechanism, have on average, of their neighbors of the second mechanism, a greater number which are in a *different* activity state, and vice-versa. (“non-correlated pairs”)

We export this original network to Cytoscape and into R. For simplicity, we reduced the number of interaction mechanisms to |*IM*|=3, filtering interactions not belonging to these mechanisms. We created two classes of interactions, where we assume interactions within each class may work in concert to achieve a biological process or molecular function. Thus, for each of our simulations, one class had two mechanisms, while the second class had only one mechanism. e.g., assume an original network has *IM*={*M*,*TR*,*B*}, class 1 is {*M*,*TR*} and class 2 is {*B*}.

Beginning with the pathway interactions, we assign interactions in the first class {*M*,*TR*} to the active state with a 90% probability, and those in the second class {*B*} to the active state with a 10% probability. From the expanded interactions, we assign interactions in the second class (e.g., B) to the active state with a 90% probability, while interactions in the first class are assigned with a 10% probability. (the “90/10” rule). This creates a network with the desired mechanism-mechanism correlations.

We created N simulations, where for simulation k, we simulated expression correlation data **r**_*k*_, using a Fisher transformation from a normally distributed random variable (see section “Noise model”), where active interaction in **I**^∗^ have a mean of 0.7, while the remaining non-active interactions have a mean of 0.

We initialized our program with a vector of initial interaction states **q**_0_, by performing a statistical test on the simulated correlation data.

To generate results for simulation k, we input **r**_*k*_ and *G* (the original network), and generated a final system realization **q**_*k*_, corresponding to a set of active interactions *A*_*k*_ in the original network. We then compare *A*_*k*_ against **I**^∗^ to evaluate the performance of the 4 algorithms in terms of sensitivity, specificity and overall percentage correct.

**Figure 3:**
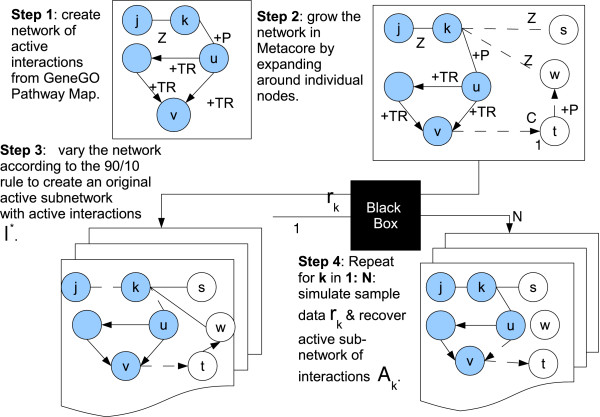
**Validation approach: Step 1: Create an original network from a canonical GeneGO Pathway Map.** These initial Pathway Interactions begin as active interactions (solid lines). Step 2: Expand the network by adding direct interaction to create new regions of Non-Pathway Interactions considered to be non-active (dotted lines). Step 3: Vary the network to create *I*^∗^ using the 90/10 rule. Notice that interactions in the class {*TR*} mostly remain active among Pathway Interactions, whereas interactions in the class {+*P*,*Z*} mostly remain active among the Non-Pathway Interactions. Step 4: Create simulation data *r*_*k*_ and recover active interactions *A*_*k*_ via the black box (algorithm under evaluation).

The validation approach is described in a step-wise process diagram in Figure 3.

#### GeneGO pathway maps used in simulations

We performed the simulation experiments using three GeneGO Pathway Maps: VEGF signaling and activation (VEGF Sig), Cell adhesion:Integrin mediated cell adhesion and migration (Cell adhesion), and Blood Coagulation. Additional files 1, 2 and 3 contain the GeneGO representation of these Pathway Maps.

We created the original active subnetworks from the GeneGO Pathway Maps using the 90/10 rule as described in “Validation using simulated correlation data for GeneGO pathway maps”. This created original networks with the following specifications. For the VEGF network: 1065 interactions (635 active) *IM*={*M*,*B*,*TR*}, active interactions remaining active with 90% probability (class 1) : {*M*,*TR*}; non-active interactions becoming active with 90% probability (class 2): {*B*}; i.e., we postulated the active subnetwork containing a region with high transcription and microRNA activity in the first instance, and region with high binding activity in the second.

For the cell adhesion network: 344 interactions (200 active); *IM*={+*P*,−*P*,*TR*}, active interactions remaining active with 90% probability (class 1): {+*P*,−*P*}; non-active interactions becoming active with 90% probability (class 2): {*TR*}; i.e., we postulated an active subnetwork containing a region with much phosphorylation and de-phosphorylation activity, and a region with regulatory interactions.

For the blood coagulation network: 94 interactions (71 active); *IM*={*C*,*B*,+*P*}, active interactions remaining active with 90% probability (class 1): {*C*,*B*}; non-active interactions becoming active with 90% probability (class 2): {+*P*}; i.e., we postulated an active subnetwork containing a region with binding activity and a region with high cleavage activity.

#### Parameter initialization

When generating results, we initialized *Ψ* as (*γ*_0_=1,*γ*_1_=1,*β*=2) for the single parameter model. For the full parameter model, we set all $\gamma_{s_{a}}$ for *s*∈*IM*,*a*∈{0,1} values to be 1, while *β*_*s*,*t*_ for *s*∈*IM*,*t*∈*IM* were set to 2 for *s*=*t* to set slight encouragement for a pair of neighboring interactions of the same mechanism to be in the same state, and to 0 otherwise.

#### Method comparison and evaluation

The single parameter approach as implemented by Wei and Li, and JActiveModules, are node based methods. They are designed to find active subnetworks in a gene or protein network when the gene expression signal is observed and is a feature of the nodes in the network. However, as described, our method is designed to find active subnetworks when a strong correlation of expression over time between two neighboring genes is observed, and this correlation is a feature of the interactions in the network. Therefore, the scoring function in our method contains an *edge-based measure*.

Therefore, to provide a fair comparison of our method to the single parameter approach, and JActiveModules, we convert their scoring function to an edge-based measure. For the single parameter approach, we simply reimplement the algorithm to create a neighborhood function over edges rather than nodes as described above in section “The MRF component”. In the case of JactiveModules, we convert the edges in the network to nodes, and nodes to edges. That is, each interaction in the original network becomes a node with a correlation score associated with that node. It is then easy enough to run JActiveModules on the new graph. For the p values, we do a statistical test on the N=5 simulated *r*_*k*_ values, using n=5 for the number of time points (samples) (as above).

In JActiveModules, we run the module finder over all p values from all N=5 simulations. We use simulated annealing as a search method with default parameter values. We switched off “regional scoring” because this feature avoids finding regions active in the case where several of a hubs genes are switched on. Our simulated dataset assumes precisely this scenario: that is, that hubs are very influential on target nodes, and therefore we want the state of one hub target to influence the state of another hub target.

We run JActiveModules module finder 3 times. For each run, we merge the resulting significant modules (modules with a *Z*−*score*>3) into a single module. We export the 3 resulting modules and load the resulting subnetworks into R for performance analysis.

When we ran the full parameter approach for the cell adhesion and blood coagulation pathways, we used only two bias parameters for *γ*_*k*_ where *k*∈{0,1} corresponding to the active and non-active states of the network. We limited the number of bias parameters to avoid overfitting, since the two networks were much smaller than the VEGF signaling network, and so do not need separate overall active state weightings per mechanism.

One simulation in each of the cell adhesion and the blood coagulation pathways resulted in runaway reinforcement where all interactions were switched to the one state. We excluded these simulations from our summary of results below. Also, one simulation did not converge in the cell adhesion pathway in the maximum number of iterations, so we excluded this simulation also. (As EM is guaranteed to converge, it would have converged with a larger maximum number of iterations.) For the blood coagulation network, we used the rank weight variation when calculating the maximum likelihood estimate for the noise function (see Section “M-step”.

Tables 2, 3 and 4 contain details of the sensitivity,specificity and percentage correct for the methods and pathways.

**Table 2:** Sensitivity of interaction finding TP/(TP+FN)

**Sensitivity TP/(TP+FN)**				
Pathway	FP-EM	SP-EM	ML	JActiveModules
VEGF signaling and activation	87.83(4.04)	83.40(2.93)	73.55(1.39)	45.63(1.30)
Cell adhesion	83.33(7.28)	78.6(5.83)	75.2(4.30)	84.83 (0.29)
Blood coagulation	94.01(2.40)	95.07(2.70)	78.87(5.27)	84.98(4.30)

**Table 3:** Specificity of interaction finding (TN/(TN+FP))

**Specificity (TN/(TN+FP))**				
Pathway	FP-EM	SP-EM	ML	JActiveModules
VEGF signaling and activation	72.37(1.85)	69.14(6.49)	72.97 (1.51)	94.60 (1.00)
Cell adhesion	84.72(7.10)	75.69(5.75)	73.19(2.23)	84.49(1.4)
Blood coagulation	64.13(4.16)	43.47(17.75)	71.30(5.83)	85.51(6.64)

**Table 4:** Percentage correct of interaction finding ((TP+TN)/total)

**Percentage correct ((TP+TN)/total)**				
Pathway	FP-EM	SP-EM	ML	JAactiveModules
VEGF signaling and activation	82.01(2.23)	78.03(0.63)	73.33(1.42)	64.10 (1.10)
Cell adhesion	83.91(3.10)	77.38(5.41)	74.36(2.78)	84.69(0.44)
Blood coagulation	86.71(1.06)	82.44(2.81)	77.02(2.97)	85.11(2.81)

When comparing the full parameter and single parameter models to the maximum likelihood approach, we must consider the noise in the simulated correlation data, which depends on the sample size n. When n is small, there is a large variation in the simulated correlation values for each interaction. In this case, the correlation statistic is less indicative of the true interaction state, and incorporating the local neighborhood activity states via the MRF component becomes more important. When n is large, the simulated correlation values more accurately reflect the activity state of interactions in the original network, and a statistical test on the correlation will provide a good indication of whether the interaction is active (regardless of neighboring interactions). In this scenario, we might expect the maximum likelihood performance will improve. However, we used n=5 in our simulations, which is a reasonable estimate of the number of samples often used in biological experiments.

For the VEGF signaling and activation network, we found that with respect to total correct interactions recovered, the full parameter approach was by far the most successful (mean of 82.01% correct), followed by the single-parameter approach (mean of 78.03% correct), followed by the Maximum Likelihood approach (mean of 74.36% correct), followed by JActiveModules (mean of 64.10% correct). On the other hand, for the Cell adhesion network, JActiveModules was slightly more successful than (but comparable to) the full parameter approach in recovering total correct interactions, with means of 84.69% and 83.91% correct respectively. Possibly, the smaller size of the network (384 interactions compared with the VEGF signaling network size of 1065 interactions), meant that there was a shortage of interactions belonging to each M-M pair used to train the model. The full parameter model was however more successful than the single parameter model and the maximum likelihood approach. Similar results are seen in the Blood Coagulation Network.

While results are comparable to JActiveModules, the use of the MRF-based approach presents additional advantages in that parameter values can be analyzed to identify mechanism correlations in the network. Further, when MRF is teamed with other tools such as a diffusion kernel [8], the accuracy may improve further, bringing advantages of both a model-based approach, efficiency and excellent accuracy.

#### Comparison of global and local feature properties of networks

We are interested in comparing how well the methods were able to recover network characteristics of the original active (or non-active) subnetwork.

We are most interested in seeing the advantage of incorporating a feature that searches for mechanism-enriched regions into active subnetwork finding algorithms. The fairest way to see this advantage is to compare characteristics of networks generated by an algorithm that incorporates this feature (the full parameter approach) to networks generated by the same algorithm without this feature (the single parameter approach). Specifically, does the full parameter approach more successfully recover topological characteristics present in the original active subnetwork than the single parameter approach? Therefore, we do not further analyze the characteristics of networks generated by JActiveModules or the Maximum Likelihood approach.

We use the Cytoscape plugin NetworkAnalyzer [41] to analyze global characteristics of the networks generated from the full parameter and single parameter approaches, and compared these to the characteristics of the original active subnetwork. We considered networks formed from interactions that were identified as active in at least 80% of the simulations (4/5).

We use the VEGF signaling and activation network to perform our analysis. Because of the way we artificially created the active subnetwork from the original network, most of the active subnetwork is made up of interactions of the binding mechanism (B), while the non-active subnetwork contains many interactions of all mechanism (B, TR and M). We therefore looked at both the active subnetworks recovered, and the non-active subnetworks recovered, which we considered to be active under other conditions and therefore still of interest.

For the active subnetworks, we first considered the betweenness centrality characteristics. The betweenness centrality (BC) of a node reflects the amount of control that this node exerts over other nodes in the network. The original active subnetwork contained a node for Entrez Gene ID 5295 (PI3K reg class IA) which had the highest BC value in the original network (BC: 0.24). The active subnetwork recovered by the full parameter approach has a BC of 0.20, while the active subnetwork recovered from the single parameter has a BC of 0.15 for the same node. The BC measure favors nodes that join dense communities, thus as expected by theory, the full parameter approach is better able to recover the dense regions of the original active-subnetwork (i.e., those dense in neighboring interactions of particular mechanisms), and is better able to recover properties of the nodes that connect these regions.

Similarly, stress centrality (SC) measures the number of shortest paths through the node. A node has a high stress if it is traversed by a high number of shortest paths. Entrez Gene ID 5295 also has the highest SC value in the original active subnetwork (SC:16335). The parameter value was recovered closely by the full parameter approach (SC:17209), while the single parameter approach network had a SC of (SC:4711) for the same node.

We also looked at the clustering coefficient (CC) of the 3 active subnetworks. The CC of a node is a measure of how well connected neighbors of a the node are. The CC distribution gives the distribution of the average of CC values for all nodes with *k* neighbors. A CC distribution that follows a strong power law (i.e., the CC value of a node drops off quickly with the number of neighbors of the node) can also indicate the subnetwork contains many dense regions, connected by individual nodes. Fitting a power law creates a linear relationship between the CC value against the number of neighbors of a node. The R-squared value measures the correlation between the two variables. After fitting a power law to the CC distribution, we found the original active subnetwork had an R-squared value of 0.775, the full parameter approach had an R-squared value of 0.646 and the single parameter approach had an R-squared value of 0.539. That is, the full parameter approach was better able to capture the modular nature of the original active subnetwork.

We then examined some characteristics of the non-active subnetwork, which contained interactions of type B, TR and M, and was thus in part a regulatory network. An important topological property of a biological network is out-degree distribution, since this can provide evidence of hubs. Analysis of out-degree distribution revealed Entrez ID 4790 (NF-kB) was the node with the largest outdegree in the network (out-degree of 31). The active subnetwork recovered using the full parameter approach has an out-degree of 35 while the single parameter approach network has out-degree 18 for the same node. While the full parameter method switched too many adjacent interactions of NF-kB to the non-active state, it achieved the out-degree value closest to the value of the node in the original non-active subnetwork. Similarly, node 407035 (microRNA 31) has out-degree 16 in the original network, 17 in the full parameter approach network, and 14 in the single parameter approach network.

Similarly, node with Entrez ID 6401 (e-selectin) has in-degree of 16 in the original active subnetwork, which is the highest in-degree in the original network. The same node has in-degree 16 in the full parameter approach network and in-degree 12 in the single parameter approach network. Thus the full parameter approach was better able to capture the nature of the multi-input regulation of e-selectin.

With regard to stress centrality (SC) parameters in the non-active network, the original network had 6 nodes in the bucket containing the nodes with the largest SC values, i.e., SC values above 10^3^, while the full parameter approach network had 5 nodes in this bucket, and the single parameter approach network had 0 nodes in this bucket. Again, the full parameter approach is best able to capture the nature of modular regions, which require the presence of “stressed” nodes, through which other nodes pass. Looking at some nodes with large SC values, c-myc (Entrez ID 4609) has SC of 3211 in the original network, SC 3234 in the full parameter network, and only SC of 735 in the single parameter network. Showing a similar pattern, c-Jun/Fos (Entrez ID 3725) has an SC of 3327 in the original network, 2162 in the full parameter network, and only SC of 243 in the single parameter network.

The Cytoscape files containing the full analysis of networks generated by both methods for both pathways are available upon request to the authors.

### Application to angiogenesis model

Hahn *et al.* previously reported on studies involving microarray-based gene expression profiling and analysis of endothelial cells plated on a well-characterized three-dimensional collagen gel model of in vitro angiogenesis (“the 3D model”) [42]. Gene expression levels were detected at times = 0.5, 3, 6, 24 hours after stimulation using microarray technology. We call this set of experiments the **“mRNA angiogenesis array”**. The mRNA angiogenesis array data is available at NCBI Gene Expression Omnibus [43] (Accession GSE779).

Subsequently, the angiogenesis 3D model experiment was repeated with a miRNA array and expression levels recorded at times = 0.5, 3, 6, 12, 24 hours (unpublished observations, Gamble JG). We call this the **“miRNA angiogenesis array”**.

We uploaded the gene list from the mRNA angiogenesis array and the miRNA angiogenesis array to Metacore and generated a network using the Build Networks tool with the Analyze network algorithm. We expanded this network to include interactions that belong to canonical Pathway Maps known to regulate the angiogenesis program. We included maps corresponding to integrin-mediated pathways because vascular integrins are essential regulators and mediators of physiological and pathological angiogenesis [44,45]. The maps used are “cytoskeleton remodeling Integrin outside-in signaling” and “Cell adhesion Integrin mediated cell adhesion and migration”. We named the network “Original Angiogenesis Network”, and imported the network into Cytoscape and R.

We then generated correlation scores for the data. As there were only four experimental time points consistent between the gene array and the microRNA array, we generated a correlation score artificially.

Specifically, we created a list of significant genes in R using the topTable command in the limma gene expression package [46] for each time point, 0.5, 3 hr, 6 hr and 12 hr (regardless of whether the gene was over or under- expressed significantly at that time point). We then generated the artificial correlation score to each interaction based on the following criteria: 

1. If neither gene was significantly expressed at any time point, score : 0

2. If at least one gene was significantly expressed at any time point: 

• •Create a binary vector for each gene specifying whether it had significant expression at times 0.5, 3 hr, 6 hr, 24 hr.

• •Perform a binary “and” operation on the two vectors.

• •Let subscore=length of the number of 1s in the result (i.e., number of common time points where the two genes either side of the interaction are co-expressed significantly).

• •Otherwise, interaction score : 0.4 + subscore/(5*2) - i.e., equally spaced between 0.4 and 0.8.

As we artificially generated correlation scores described above, the application of our algorithm to this dataset merely gives us an indication of which interactions are active, and cannot be considered to be a statistical application.

We ran the single parameter and the full parameter algorithms on the Original Angiogenesis Network using the artificial correlation scores. We identified interactions recovered as active under the full parameter but not the single parameter approach (“FP extra interactions”), and those recovered as active under the single parameter but not the full parameter approach (“SP extra interactions”). We uploaded the full set of active interactions, and the extra interactions, for both algorithmic approaches to Metacore for further analysis. There were 12 FP extra interactions and 9 SP extra interactions in total.

Many of the interactions recovered by the two methods appeared in signaling cascades common to GeneGO Pathway Maps that are active during the angiogenesis process. The relevant GeneGo Pathway Maps contain pathways that regulate the cell adhesion process via the integrins. The GeneGO Pathway Maps we considered are: Cell Adhesion - ECM Remodeling; Cell Adhesion-Integrin-mediated adhesion and migration, and Cytoskeleton Remodeling-Integrin Outside-In Signaling. Integrins are heterodimeric adhesion receptors composed of alpha- and beta-subunits. It is known that at least 18 distinct alpha subunits and 8 or more beta subunits lead to generation of 24 alpha/beta heterodimeric receptors. Most integrins recognize extracellular matrix (ECM) proteins, such as Laminin, Fibronectin, Vitronectin and Collagen (types I, II and IV) [47].

Looking at the FP extra interactions, only the full parameter approach recovered the interaction “ITGB B Talin” (beta integrin unit binding to Talin). Talin binding to integrin beta tails is a common element of the signaling cascades in the aforementioned Pathway Maps that control integrin activation. The binding step causes the integrin receptors to change conformation and increase binding affinity to ECM proteins. Collagen binding to integrins is part of the cytoskeleton remodeling. While both full parameter and single parameter approaches detected binding of Collagen II to alpha-2/beta-1 integrin, only the full parameter approach detected Collagen IV binding to the same integrin.

Looking further downstream in the Cytoskeleton Remodeling-Integrin Outside-In Signaling pathway, both approaches failed to recover the phosphorylation of MEK1(MAP2K1) and MEK2(MAP2K2) by c-Raf-1, or the subsequent phosphorylation by both kinases of ERK1/2. This cascade leads to the activation of the c-Jun/c-Fos complex transcription factor that is required for cell proliferation. However, the full parameter approach did detect Tcf(Lef) activation of Cyclin-D1, which is required for cell cycle activation. There were several additional transcription regulation (TR) interactions detected for Tcf(Lef) in the full parameter method but not the single parameter method, which is consistent with the large parameter value learned for M-M pair “TR-TR” (5.04). The activation of Tcf(Lef) together with the learned strong correlation for TR-TR, indicating such interactions tend to be co-regulated, meant that other Tcf(Lef) target interactions were detected including SOX2 and hsa-let-7c.

Further, only the full parameter method detected the interaction *ARP*2/3→*ActinCytoskeleton*, which is required to activate actin polymerization [48]. Only the full parameter method recovered the interaction *CRK*→*RAC*1. Activation of Rac1 leads to membrane ruffles, formation of lamellipodia and cell migration [49]. The only interaction recovered by the single parameter approach in the cell adhesion and cytoskeleton remodeling pathways was GSK3 activation of beta-catenin. In all, the full parameter approach was more successful at recovering interactions within this group of GeneGO Pathway Maps, while the extra interactions recovered by the single parameter approach do not belong to any particular pathway. The large value for M-M pair parameter for “B-B” (2.63) meant the full parameter approach had a tendency to keep the binding interactions which characterise integrin binding to ECM proteins in the same state as one-another, allowing recovery of more active binding interactions in this pathway.

Recent evidence suggests the miR-29 family consisting of miR-29a, miR-29b and miR-29c are antiangiogenic [50]. Both approaches recovered miR-29 targeting of beta integrin subunit (ITGB1) in the active subnetwork. The Original Angiogenesis Network also contained an interaction from the GeneGO database showing miR-29b targeting of collagen IV gene COL4A1 [51,52]. This interaction was not recovered by either the single parameter or full parameter approach. We might have thought the full parameter approach would detect this interaction, since miR-29b is already considered to be active in its role in inhibiting the pro-angiogenic integrins. However, in this situation, there was no evidence of gene expression of the collagen genes in our input gene list, and thus possibly the low artificial correlation score (the noise component) was more influential than the local neighborhood influence (MRF component) in this circumstance.

**Figure 4:**
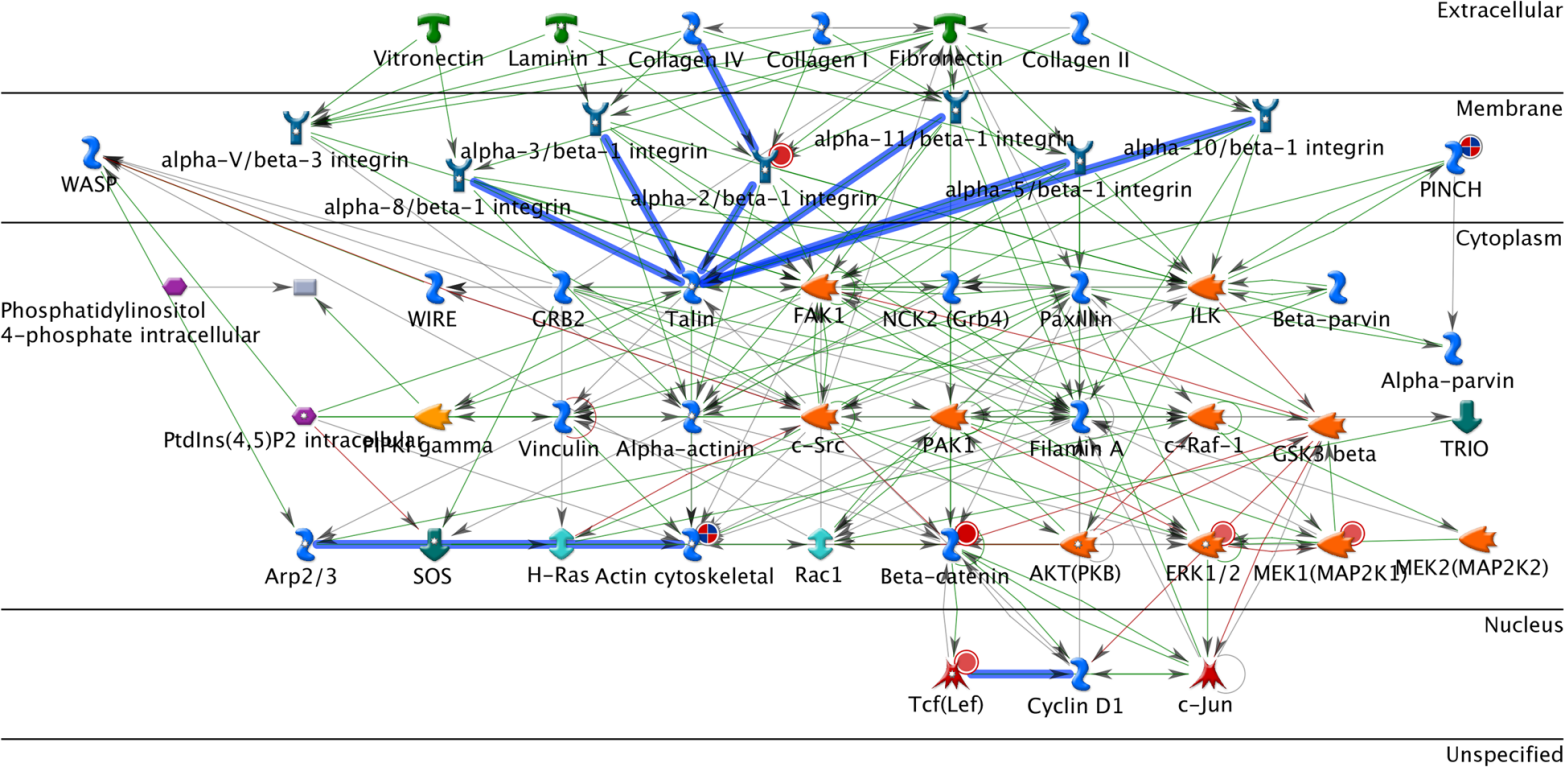
A network showing the interactions from GeneGO pathway map: cytoskeleton remodeling - integrin inside out signaling, overlaid with the extra interactions detected using the full parameter approach.

Figure 4 contains the interactions from GeneGO Pathway Map: Cytoskeleton Remodeling - Integrin Inside Out Signaling, overlaid with the extra interactions detected using the full parameter approach.

### Application to mouse organogenesis data

In order to test our method using actual Spearman correlation values in the likelihood function, rather than our artificial scoring system used on the angiogenesis data, we applied the method to data from a developmental mouse lung organogenesis study [53], downloaded from NCBI Gene Expression Omnibus (GEO) [43], Accession GSE20954 and GSE21052. As the experiments were performed using Affymetrix chips with known 45,000 probes (mRNA data) as opposed to the home made chips, we had expression data matching nearly all Network Objects in the Metacore networks, not only the so-called seed nodes. Secondly, we had a stronger signal when calculating Spearman correlation coefficients across interaction expression data.

To create a set of mRNA and miRNA genes to upload to Metacore in our list we wished to identify miRNAs and mRNAs that were expected to regulate the same processes. In particular, we wanted late-onset genes that had high expression in the adulthood stage, so we could compare our own biological analysis of resulting networks with the biological analysis of similar gene sets in the original network. In this work, cluster 6 mRNAs and cluster 1 miRNAs were considered to be late onset genes. For the miRNA array, we used the clusters generated by [53] (provided to us by the authors) and selected miRNA cluster 1, which also had a peak late onset adulthood among member miRNAs.

To closely reproduce their results, we clustered the 11220 mRNA probes identified as active in [53]. We selected a subset using decideTests in the limma package in R in the contrast PN30.adult-PN10 as active. We then clustered the Affymetrix probe expression values over the 7 time points using hierarchical clustering in R (hclust). We created a representative expression profile for each cluster by finding the mean expression value of all probes in the cluster at each time point (see Additional file 4). Among our clusters, the representative profile for cluster 2 (containing 850 probes) showed a peak at the late adulthood time. We converted the probe IDs to Entrez Gene IDs using tools in the package GEOQuery [54] and limma [46]. We then input the two sets of late onset genes for the miRNA and mRNA clusters into Metacore and used the Build Networks tool with the “Analyze network” algorithm to create a network in Metacore, which we called “Original Mouse Lung Organogenesis (MLO) Network”.

Some difficulties arose in creating a correlation score for each interaction because Metacore network interactions are between Network Objects which map to multiple Entrez Gene IDs, which in turn map to multiple Affymetrix probe IDs. As the expression data is matched to Affymetrix probe IDs, some choice was required when selecting which probes’ expression data should be used to create a correlation score for an interaction between two Network Objects. First, we created a correlation matrix for the Entrez IDs, and where there were multiple probes matching one Entrez Gene ID, we selected the probe, which had the largest mean correlation score against each other probe in the network. That is, we calculated the probes correlation with all the other probes matching genes in the network, and selected the probe with the largest mean value to represent the Entrez ID. When creating a correlation vector to match the interaction between two Network Objects, where there were multiple Entrez Gene IDs matching the Network Object, we took the mean of the correlation values to represent the interaction.

We exported the Original MLO Network from Metacore into Cytoscape and into R. The resulting network had 99 unique nodes and 106 interactions. We imported the correlation data into R and ran the single and full parameter approaches on the data, using the rank weight estimate. We adjusted the noise model slightly, since we now had correlation values <0, and so we tested the absolute value of the correlation coefficient.

Both the full parameter and the single parameter approaches found some interactions present in the Cytoskeleton Remodeling - Keratin Filaments Pathway Map. This map shows the components of the intermediate filaments cytoskeletal system, that interact with actin and microtubule filament systems to control cell assembly. Binding proteins such as Plectin 1 and Desmoplakin coordinate the interconnection of the three filament systems by binding to filament systems. For example, Plectin 1 binds to Keratin 5/14 complex in the Pathway Map and the Metacore network [55]. Kinases including CDK1 (p34) regulate the activity of these binding proteins and IFs. In particular, only the full parameter approach recovered CDK1(p34) inhibition of Plectin 1 [56] by phosphorylation and also via inhibition of GRB2 which binds to Plectin 1 [57]. These interaction are present in the Cytoskeleton Intermediate Filaments process network and the Keratin Pathway Map.

The full parameter approach also switched on a cluster of phosphorylation interactions by the two kinases TRPM6 and TRPM7 of the myosin superfamily, including MYH14, MYH9 and MYH10. All three myosin Network Objects were found to be regulated by both kinases. Myosins are actin-based motor proteins that function in the generation of mechanical force in eukaryotic cells. Non-muscle myosin heavy chain proteins (MYH9, MYH10, MYH14) are involved in control of cytokinesis, cell motility and maintenance of cell shape. The three myosins are a group relation of MYHC (see in the figure). MYHC functions in skeletal muscle contraction. Interestingly, smooth muscle contraction was found to be an over-regulated category for cluster6 (late onset mRNAs) in the study of Dong *et. al*, and thus this finding is consistent with the findings in the original study.

The single parameter approach only detected the interaction TPRM6 phosphorylation of MYH14, while as mentioned the full parameter approach recovered 7 phosphorylation interactions of the myosin proteins in this cluster. The reason for the more successful detection of the phosphorylation of the myosin proteins by the kinases can be found by examining the parameter for mechanism-mechanism pair {+*P*,+*P*} which was found to be the moderately high value of 0.85.

In the Original MLO Network, there were many additional miRNA interactions recovered as active in the full parameter approach, that were not recovered by the single parameter approach. These interactions are: “microRNA 30b” → “LIN-28” (“M”), “microRNA 26b” → “CPEB2” (“M”), “microRNA 223” → “Galpha(q)-specific peptide GPCRs, CCR1” (“M”), “microRNA 223” → “PLEKHM1” (“M”), “microRNA 34b ∗” → “WNT3” (“M”), “microRNA 34b ∗” → “Rab-3” (“M”), “microRNA 667” → “MYH14” (“M”) and “microRNA 24-1 ∗” → “TTLL4” (“M”). The mechanism-mechanism parameter for the {*M*,*M*} pair is 0.75, again a moderately high value.

A potential interaction worth further exploration is the regulation by microRNA 24-1 ∗ of TTLL4, which is a polyglutamylase that preferentially modifies beta-tubulin and therefore regulates the formation of microtubules that control cytoskeleton stability and dynamics. A further interaction of interest is miRNA 34b ∗→ WNT3, as the p53-miR34 ∗ network is known to regulate the canonical Wnt signaling pathway in organ development and human cancer [58].

Some of the interactions annotated in Metacore of mechanism “M” are supported by sequence based predictions and are therefore low trust interactions. They do not appear in GeneGO Pathway Maps or other canonical pathways. For example, “microRNA 667” → “MYH14” (“M”) and “microRNA 24-1 ∗” → “TTLL4” (“M”) are both low trust interactions in the GeneGO knowledge base, but are recovered using the full parameter approach. By highlighting low trust interactions using the full parameter approach, we are able to guide the user to possible new interactions active in their data which regulate known processes and pathways.

**Figure 5:**
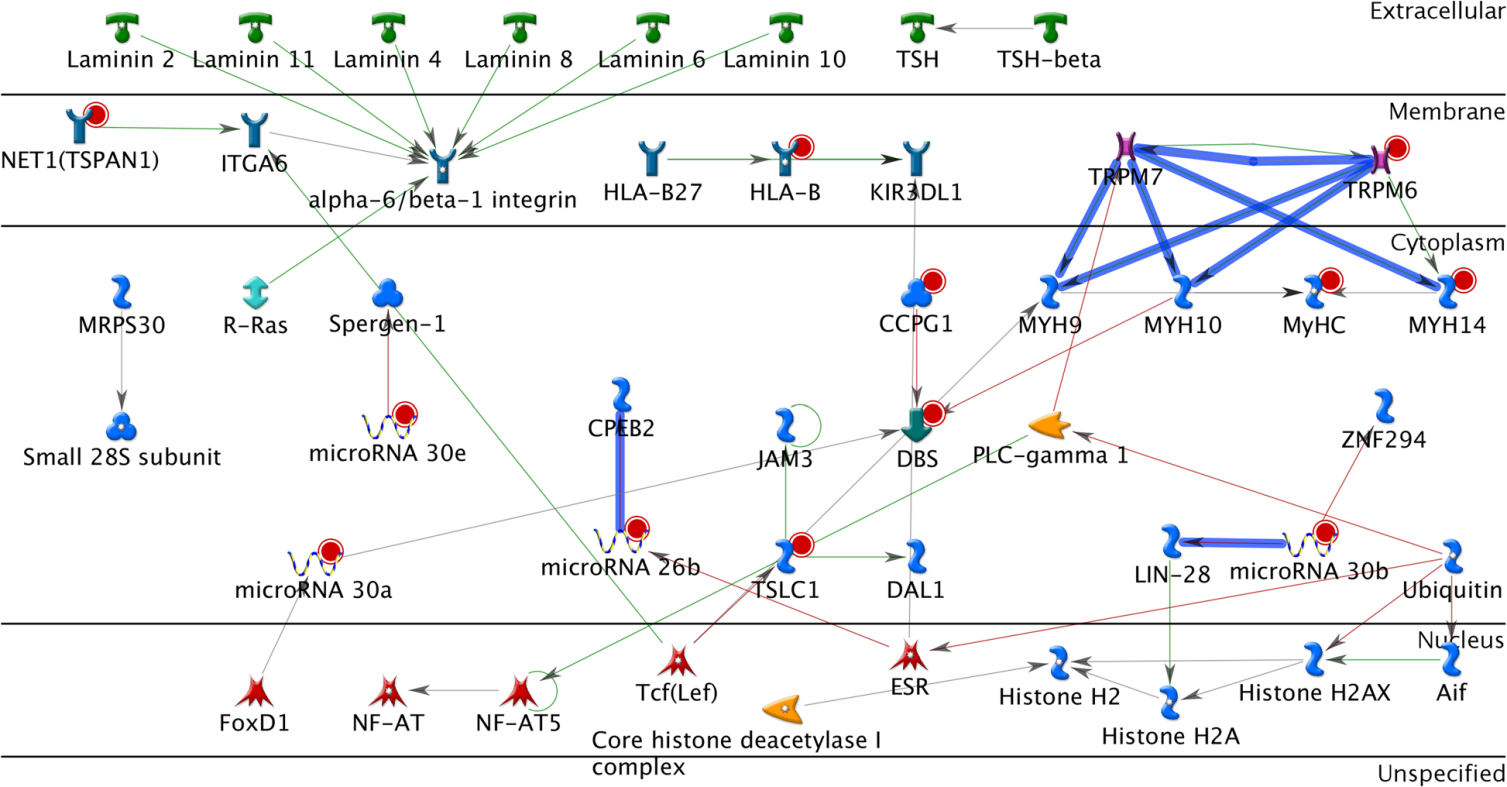
**A network showing several interactions from original mouse lung organogenesis network created in Metacore, overlaid in blue with the extra interactions detected using the full parameter approach, but not the single parameter approach.** This region of the network shows the cluster of phosphorylation of myosin proteins not detected using the single parameter approach.

Figure 5 shows interactions in the Original MLO Network identified as active using the full parameter approach, which were not identified as active using the single parameter approach.

## Conclusions

We developed and implemented a method to find active subnetworks in a condition from an original network generated by Metacore. Our method is unique in that it learns the value of distinct parameters that express the relationship between a pair of interactions based on the two respective mechanisms of the pair. We compared our method to a previously published method, which was a simpler, more general, method using only a single parameter to express such dependencies, as well as to the popular active subnetwork recovery tool JActiveModules, and a straightforward maximum likelihood tool.

Our method was validated against these methods using original networks generated from GeneGO canonical Pathway Maps. We found our method have better performance than these three methods among networks meeting certain criteria, which we describe as M-M Pair Rich Active Subnetworks (described in section “M-M pair rich active subnetworks”). We applied our method to two sets of experimental data which both consisted of matching mRNA and microRNA time-course expression data. We were able to find interactions belonging to biological pathways consistent with the experimental study in the full parameter approach, that were not detected with the single parameter approach.

### Advantages of the full parameter approach

The full parameter method has several advantages over the single parameter approach. First, it is an edge-based method (rather than a node-based method) which gives it some advantages over node-based methods. Node-based methods do not provide information as to which interactions from the original network participate in the condition. This is typically inferred by connecting the active nodes [1]. Edge-based methods provide additional information regarding which paths are active between nodes, and therefore describe exactly how the active subnetwork executes its functions. Second, analyzing a set of active nodes can provide an understanding of enriched functional categories of genes [59]. However, if we accurately know the active interactions, we can analyze network properties such as global topology properties, and local properties such as the presence of functional local components (motifs).

The second advantage is that we apply a heuristic-EM algorithm for this probabilistic problem with a latent variable approach, which is a superior optimization method to the method invoking the ICM inference algorithm used in [35]. Third, as emphasized, we incorporate multiple finely-tuned mechanism-based parameters in the scoring function. Indeed, our literature search suggests we are the first to design an active subnetwork scoring function that incorporates a neighborhood influence factor that varies according to a neighbor’s biological type or function.

#### Advantages of using M-M Pair correlation parameters

The incorporation of multiple parameters to model mechanism-mechanism pair effects provides many benefits. The first, is the potential for greater predictive accuracy so long as the complexity of the multi-parameter model does not lead to over-fitting. This method will have the greatest predictive accuracy in recovering active subnetworks in M-M Pair Rich Active Subnetworks. Our method when applied to such networks will recover a wide range of parameter values, with larger values expected for pairs with corresponding I-I interactions that occur in the same region of the active subnetwork.

A second benefit of using the model-based approach is the parameters representing the mechanism-mechanism correlations provide insight into the biological function of the active subnetwork. If the active subnetwork is responsible for carrying out a biological function (e.g., adhering cells in the creation of new blood vessels), this function may broken up into parts (e.g., regulatory, signaling, protein binding), where those parts are executed through regions containing only a small set of all possible mechanisms. For example, in the Original Angiogenesis Network, a large parameter value for {*TR*−*TR*} pair (5.04) cluster of neighboring Transcription Factor interactions outgoing from Tcf(Lef) were found to be active by the full parameter approach. This suggests the existence of a region of the network responsible for carrying out a regulatory function.

### Limitations and future work

There are several limitations to our approach. One limitation is that increasing the number of parameters may lead to overfitting in cases where the network only has a small number of I-I pairs corresponding to the mechanism-mechanism pair for that parameter. This can be overcome by filtering the network to only include interactions belonging to mechanism-mechanism pairs that have a minimum number of corresponding I-I pairs in the network.

Another limitation is that our noise model currently relies on calculating the similarity of gene expression between two components as a measure of the likelihood of an interaction, regardless of its mechanism. Some interactions belong to mechanisms which occur at the protein level, and it would be more appropriate to use other data sources such as protein-protein interaction data, or binding assay data. For other interactions which occur at the transcription level, RNA sequence or array data is perhaps more appropriate.

A further limitation of our work is that it assumes that the genes matching interacting Network Objects will be positively or negatively correlated in their differential expression (i.e., activated and inhibited together) in the one condition, regardless of the annotated effect of that interaction (inhibitive or activating). This can be improved in future versions of our algorithm, by requiring a positive correlation for activating interactions and negative correlation for inhibitive interactions. Future work could also model expected expression behavior of genes in simple 3-node network motifs, where such motifs are properly annotated, such as in GeneGO Metacore. For example, competitive binding versus co-operative binding could be modeled. Competitive interaction between two transcription factors, may mean that when one factor is active, the other is less likely to be active. This would improve accuracy over our current model which treats all neighboring transcription factor - target interactions equally.

Finally, a problem lies in that the algorithm currently learns parameters expressing a correlation between mechanism-mechanism pairs across the entire network. Sometimes, correlations differ according to context, i.e., subcellular localization, regional function etc. A future improvement may limit the use of global parameters to local regions, i.e., only apply a parameter for B-B mechanisms to a region of the network identified as protein binding.

This work is a first attempt at learning global rules expressing correlations between mechanism-mechanism pairs, and future fine-tuning of this work would be a welcome extension.

## Competing interests

The authors declare that they have no competing interests.

## Authors’ contributions

IL conceived the idea of finding active subnetworks enriched in a set of mechanisms by exploiting the controlled vocabulary in Metacore. TC and IL designed the probabilistic graphical model and factor graph. MC and IL planned the validation approach and application of the model to two separate experimental data sets. JG and MV were responsible for the acquisition of the angiogenesis data sets. IL implemented the program in R, performed the simulations, validation, and application to the experimental data sets, and prepared the manuscript. All authors have revised the manuscript and given approval for publication.

## Supplementary Material

Additional file 1 GeneGO Pathway Map: VEGF Signaling and Activation. Click here for file

Additional file 2 GeneGO Pathway Map: Cell Adhesion and Migration. Click here for file

Additional file 3 GeneGO Pathway Map: Blood Coagulation. Click here for file

Additional file 4 **Clusters of mRNA Affymetrix probes.** Clusters of 11220 mRNA Affymetrix probes from mouse organogenesis data set filtered by those with a significant contrast from PN30-PN10. The y-axis shows the mean expression value of all probes in the cluster, at each time point (x-axis). Click here for file
