# Supplementary figures and images for: Active subnetwork recovery with a mechanism-dependent scoring function; with application to angiogenesis and organogenesis studies
Source: BMC Bioinformatics. 2013 Feb 21;14:59. doi: 10.1186/1471-2105-14-59 (PMC3663784; doi:10.1186/1471-2105-14-59)

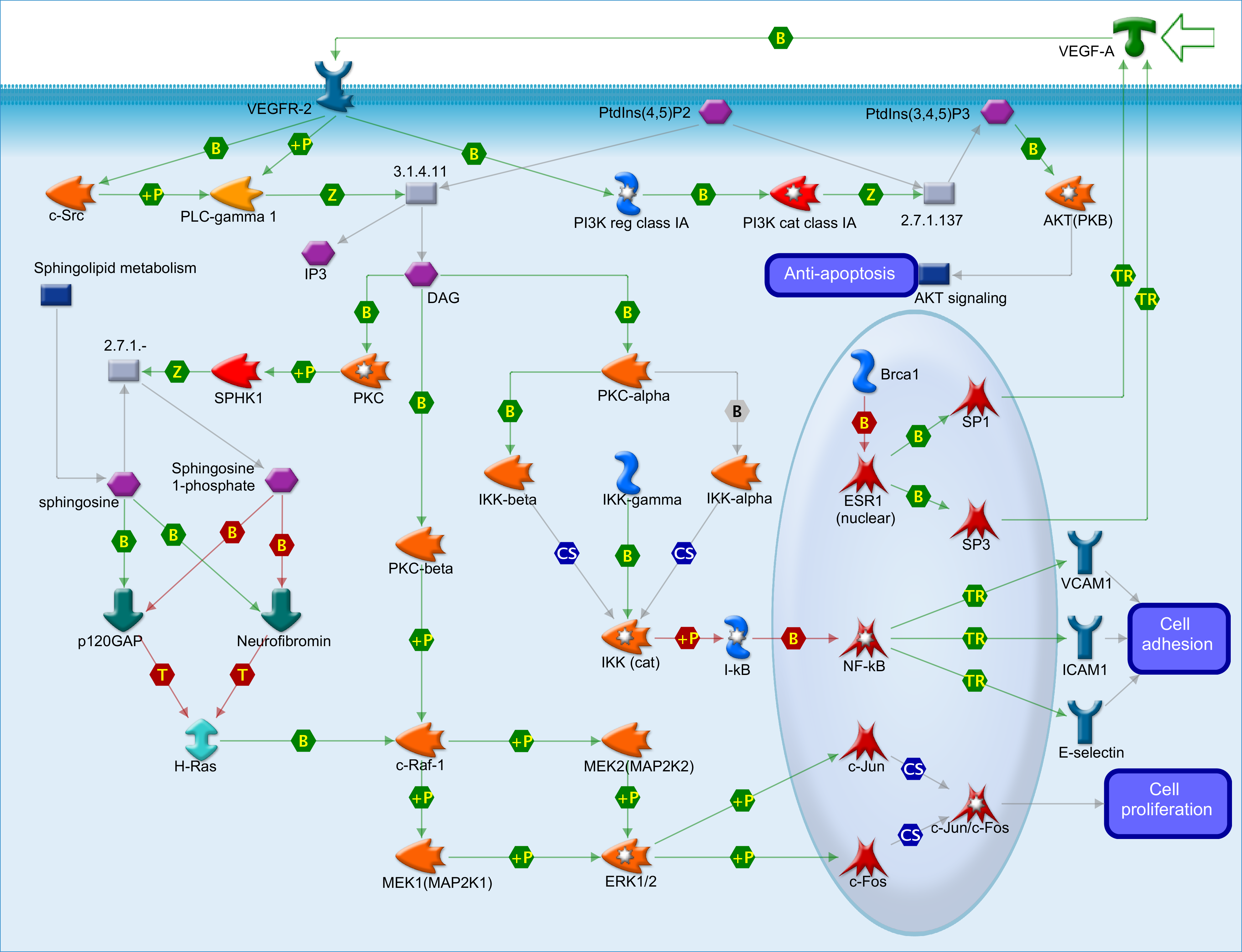

Supplement: Additional file 1 — GeneGO Pathway Map: VEGF Signaling and Activation. [file 1471-2105-14-59-S1.png]

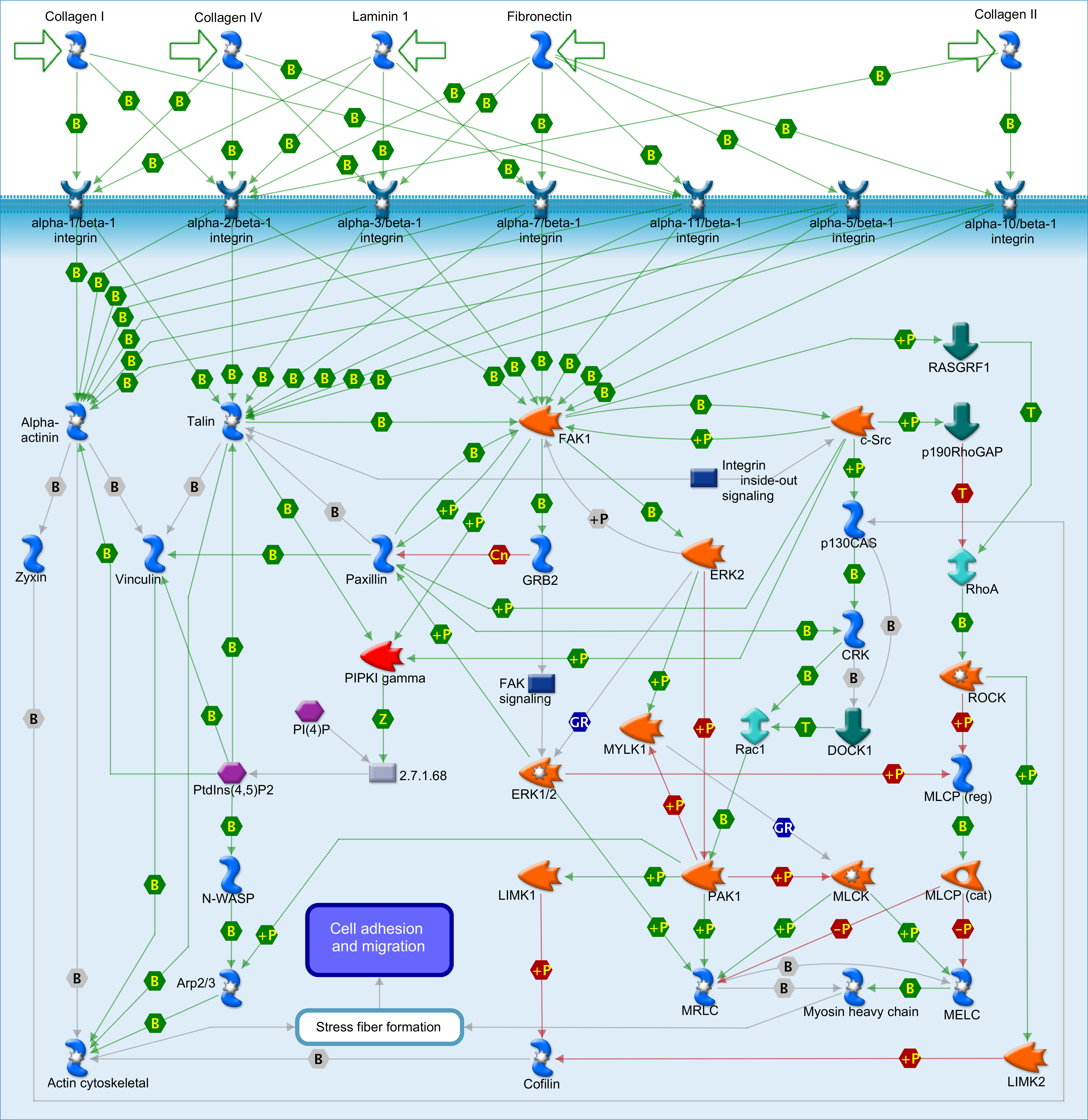

Supplement: Additional file 2 — GeneGO Pathway Map: Cell Adhesion and Migration. [file 1471-2105-14-59-S2.png]

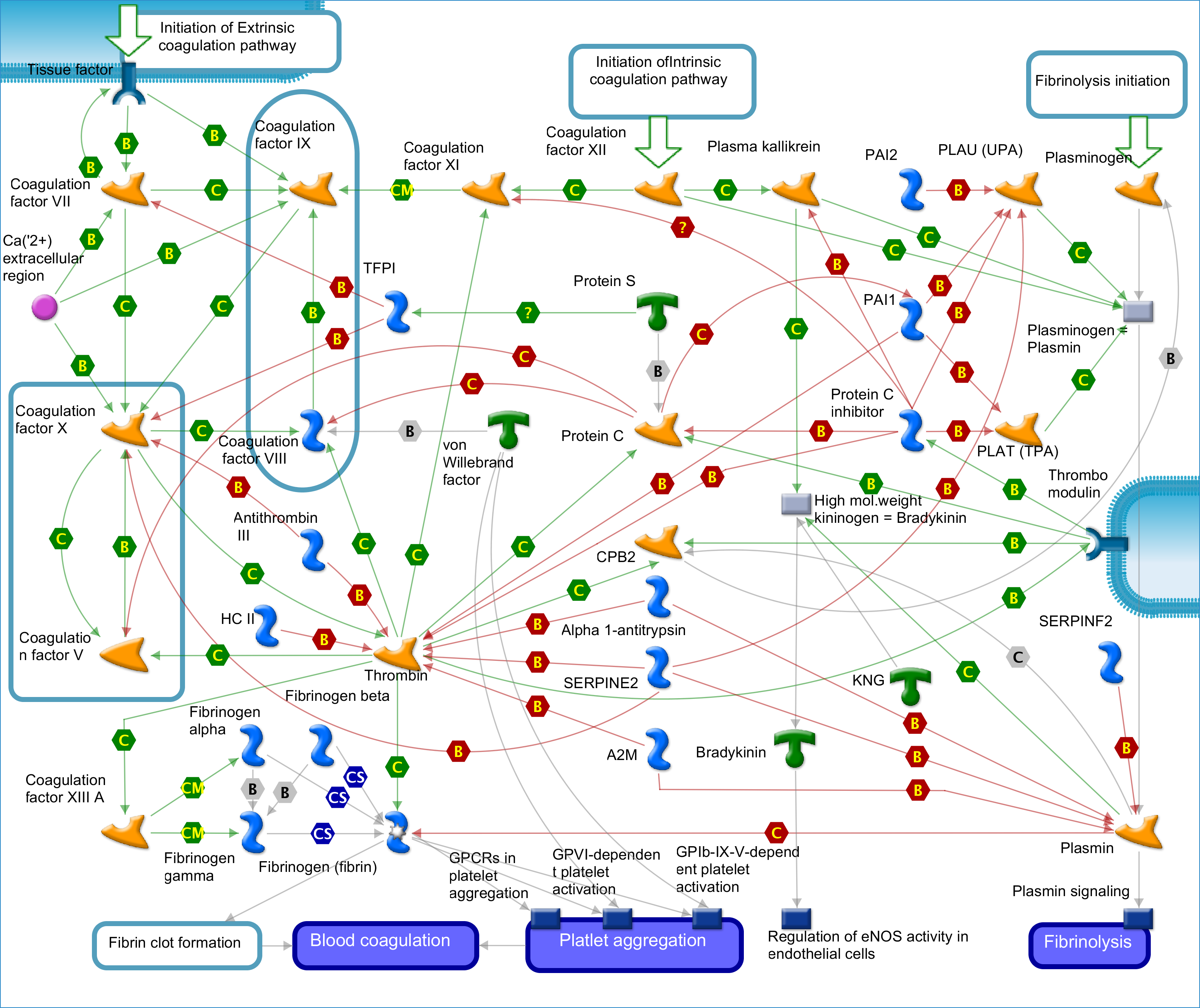

Supplement: Additional file 3 — GeneGO Pathway Map: Blood Coagulation. [file 1471-2105-14-59-S3.png]

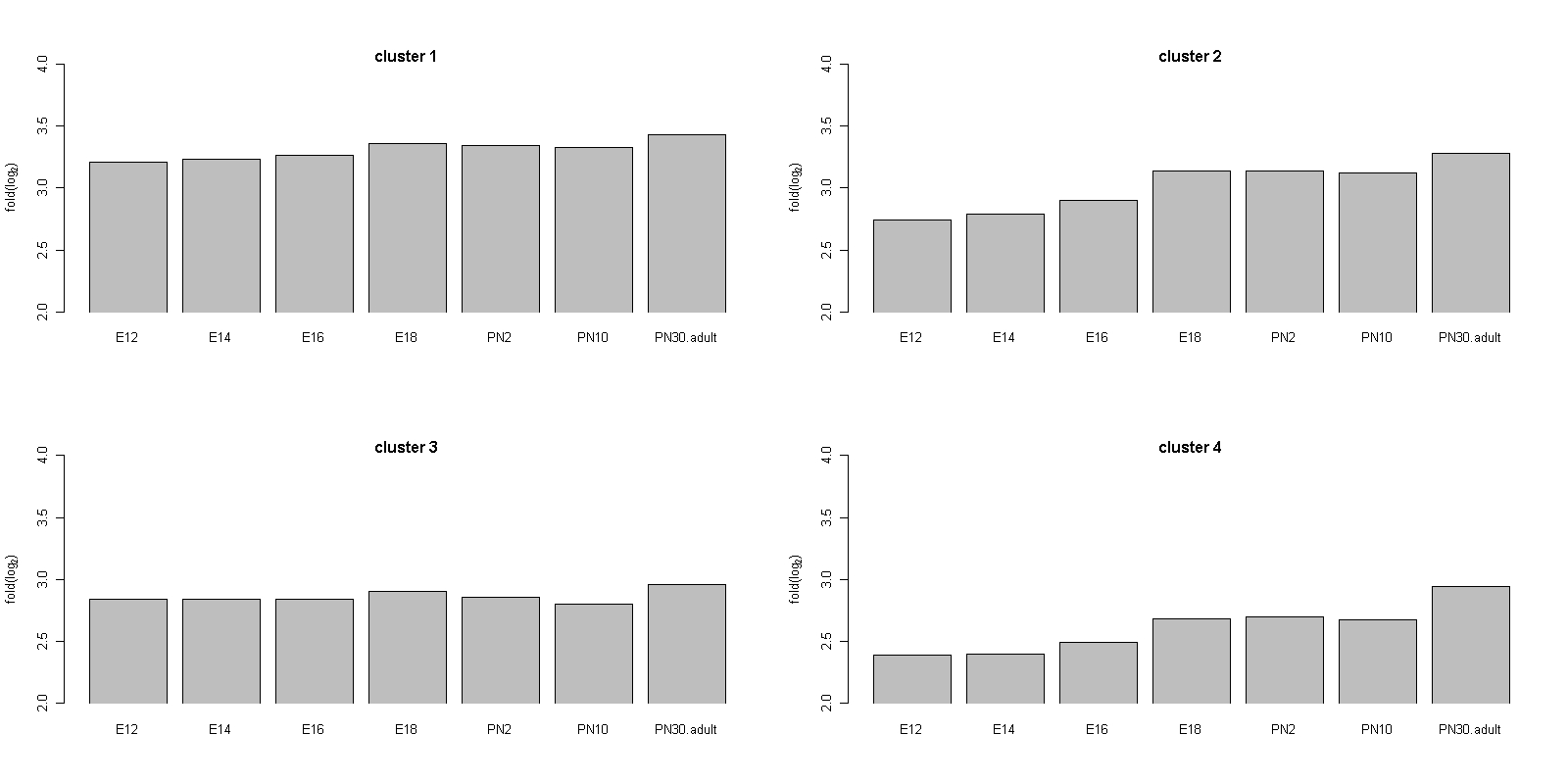

Supplement: Additional file 4 — Clusters of mRNA Affymetrix probes. Clusters of 11220 mRNA Affymetrix probes from mouse organogenesis data set filtered by those with a significant contrast from PN30-PN10. The y-axis shows the mean expression value of all probes in the cluster, at each time point (x-axis). [file 1471-2105-14-59-S4.jpeg]
